# Therapeutic targeting of the conserved region within the low-complexity domain of TDP-43 is neuroprotective and extends survival in amyotrophic lateral sclerosis mice

**DOI:** 10.1038/s43587-026-01166-3

**Published:** 2026-07-03

**Authors:** Ju Gao, Devanshi Shukla, Mao Ding, Siyue Qin, Fan Tang, Evelyn Guerrero, Lauren Vicuna, Jiawei Xu, Hongling Li, Masaru Miyagi, Pan P. Li, Jingjing Liang, Xinglong Wang

**Affiliations:** 1 Department of Pharmacology and Toxicology, College of Pharmacy, University of Arizona, Tucson, AZ USA; 2 Department of Psychiatry and Behavioral Sciences, Division of Neurobiology, Johns Hopkins University School of Medicine, Baltimore, MD USA; 3 Department of Pharmacology, Case Western Reserve University, Cleveland, OH USA; 4 Department of Pharmacy Practice and Science, College of Pharmacy, University of Arizona, Tucson, AZ USA

**Keywords:** Neurodegeneration, Neurodegenerative diseases, Ageing

## Abstract

Autosomal dominant mutations in *TARDBP*, encoding TAR DNA-binding protein 43 (TDP-43), cause amyotrophic lateral sclerosis (ALS), and TDP-43 pathology is a hallmark of multiple aging-associated neurodegenerative diseases. Despite its pathological role, effective therapies remain limited by the lack of safe, potent molecules targeting TDP-43 neurotoxicity. Here we show that the conserved α-helical region spanning residues 320–340 (conserved region or CR) is a therapeutically actionable target for TDP-43 neurotoxicity. Deletion of CR markedly suppressed TDP-43-induced neuronal death. Structure-based virtual screening identified XL20, a brain-penetrant small molecule that engages CR and confers neuroprotection without affecting TDP-43 splicing activity. XL20 alleviated motor neuron loss, extended survival in TDP-43 p.Ala315Thr ALS mice and enhanced neuronal function in p.Gln331Lys induced pluripotent stem cell-derived human ALS motor neurons. Mechanistically, targeting CR suppressed TDP-43 mitochondrial localization and restored mitochondrial function, likely through liquid–liquid phase separation. Our findings highlight CR as a therapeutic target for TDP-43-associated neurodegeneration and support CR-binding small molecules as therapeutic candidates.

## Abstract (web summary)

Gao et al. identify a toxicity-driving region within TDP-43 and show that a brain-penetrant small molecule targeting this region improves mitochondrial function, reduces neurodegeneration and extends survival in ALS mouse models.

## Main

Neurodegenerative diseases are characterized by progressive neuronal loss and aberrant protein aggregation in the central nervous system (CNS). Among the pathological proteins implicated in these diseases, TAR DNA-binding protein 43 (TDP-43) has emerged as a major contributor to amyotrophic lateral sclerosis (ALS) and frontotemporal lobar degeneration with TDP-43 pathology, with autosomal dominant mutations in the gene encoding TDP-43 (*TARDBP*) identified in both familial and sporadic forms of ALS^1,2^. Furthermore, TDP-43 pathology characterized by nuclear depletion and cytoplasmic accumulation of detergent-resistant, ubiquitinated or hyperphosphorylated TDP-43 inclusions in the cytoplasm, is a hallmark of both ALS and frontotemporal lobar degeneration with TDP-43 pathology^1,2^. These TDP-43 pathological manifestations were also observed in many other neurodegenerative diseases, including Alzheimer’s disease^3,4^ and Alzheimer’s disease-related dementias such as limbic-predominant age-related TDP-43 encephalopathy and cerebral age-related TDP-43 with sclerosis^5^, highlighting TDP-43 as a potential common therapeutic target.

TDP-43 predominantly resides in the nucleus through its nuclear localization signal (NLS), and contains an N-terminal domain, two RNA recognition motifs (RRM1 and RRM2) and a carboxy-terminal glycine-rich low-complexity domain (LCD) to mediate its primary function in messenger RNA (mRNA) splicing, transport and translation^6–11^. Although TDP-43 has been proposed to contribute to neurodegeneration through both toxic cytoplasmic aggregation and nuclear loss of function, increasing evidence supports cytoplasmic aggregation as a key mechanism driving both cytoplasmic gain of toxicity and nuclear loss of function^12,13^. Nevertheless, as the understanding of TDP-43 targets expands, there is growing interest in therapeutic interventions to address different aspects of TDP-43 pathology^14^. Small molecules offer advantages in drug development due to their ease of absorption, distribution, metabolism and excretion, along with their potential for optimization to enhance efficacy and safety. However, few small molecules targeting TDP-43 have progressed to clinical phases, and there is an urgent need for safe, blood–brain barrier-permeable molecules that selectively suppress pathological TDP-43 neurotoxicity while preserving its essential nuclear RNA-processing functions.

## Results

### TDP-43 neurotoxicity depends on conserved region

**Fig. 1:**
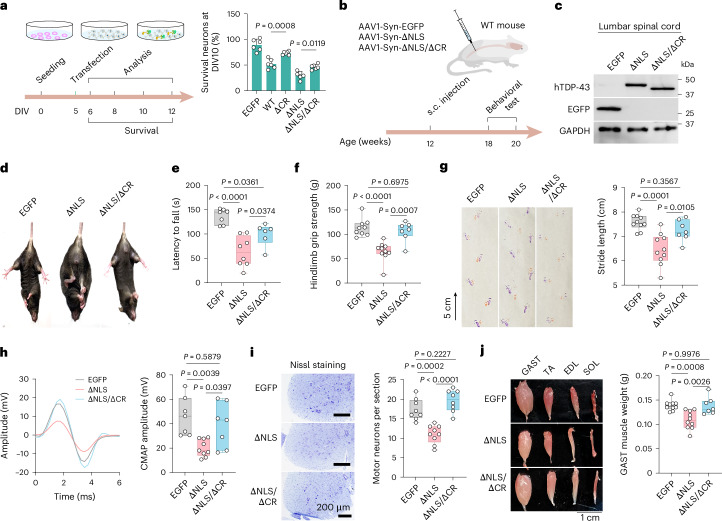
CR deletion suppresses TDP-43 neurotoxicity. **a**, Schematic of primary neuron culture (left) and survival analyses of mouse primary neurons expressing EGFP, EGFP–TDP-43 WT, ΔCR, ΔNLS or ΔNLS/ΔCR at day in vitro 10 (DIV10; *n* = 6 independent neuronal cultures). **b**, Schematic of lumbar spinal cord injection of AAV1-Syn-EGFP, ΔNLS or ΔNLS/ΔCR in WT mice. Syn, synapsin-1 promoter; s.c., subcutaneous. **c**–**j**, Representative immunoblots of human TDP-43 (**c**), tail suspension performance (**d**), rotarod analyses (**e**; *n* = 7 EGFP, 8 ΔNLS and 6 ΔNLS/ΔCR mice), hindlimb grip strength (**f**; *n* = 9 EGFP, 10 ΔNLS and 7 ΔNLS/ΔCR mice), representative footprint analyses and stride length quantification (**g**; *n* = 10 EGFP, 10 ΔNLS and 7 ΔNLS/ΔCR mice), representative compound muscle action potential (CMAP) recordings and quantification following sciatic nerve stimulation (**h**; *n* = 7 EGFP, 9 ΔNLS and 7 ΔNLS/ΔCR mice), representative spinal cord images and motor neuron quantification (**i**; *n* = 8 EGFP, 9 ΔNLS and 8 ΔNLS/ΔCR mice) and representative skeletal muscle images and gastrocnemius (GAST) muscle weight quantification (**j**; *n* = 10 EGFP, 10 ΔNLS and 7 ΔNLS/ΔCR mice) from 5-month-old WT mice injected at 3 months of age. TA, tibialis anterior muscle; EDL, extensor digitorum longus muscle; SOL, soleus muscle. Representative images and immunoblots were reproduced in at least three independent experiments with similar results. Data are presented as the mean ± s.e.m. Box-and-whisker plots show the median, interquartile range and min–max values; dots indicate individual data points. Statistical analyses were performed using one-way analysis of variance (ANOVA) followed by Tukey’s multiple-comparison test. Source data

**Extended Data Fig. 1:**
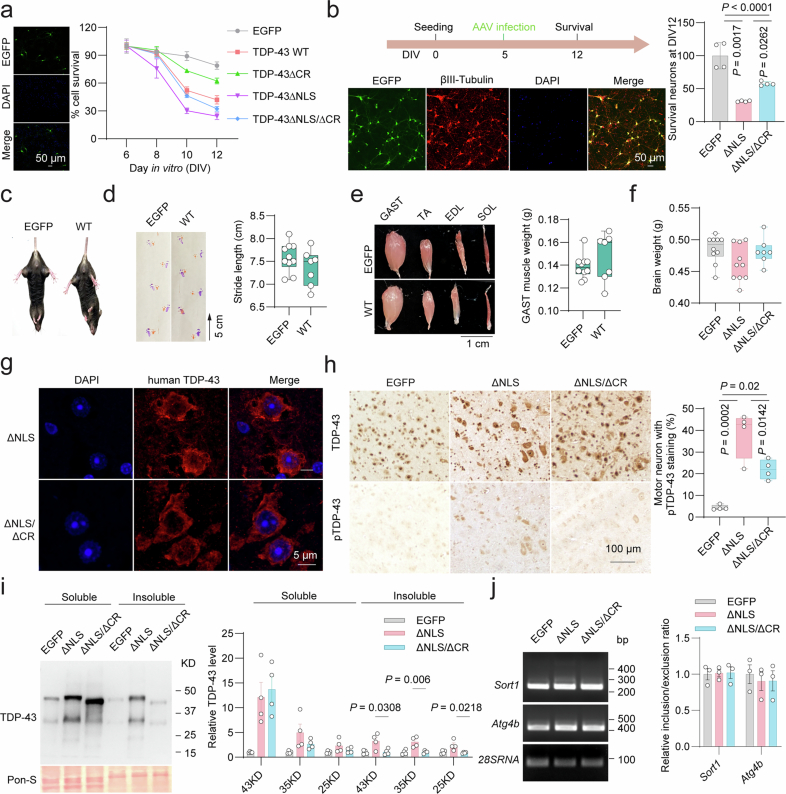
CR deletion suppresses TDP-43-induced neuronal toxicity. (**a**) Representative images and survival quantification of transfected primary neurons from DIV6 to DIV12. Transfection efficiency was ~20–30% (*n* = 6 independent neuronal cultures). (**b**) Schematic of primary neuron culture and AAV1 infection timeline (top). Representative images and survival quantification of neurons infected at DIV12. Infection efficiency was ~100% (*n* = 4 independent neuronal cultures). (**c**) Representative tail suspension performance of AAV1-Syn-EGFP- and AAV1-Syn-TDP-43-injected mice. (**d**) Representative footprint analyses and stride length quantification (*n* = 10 EGFP and 7 TDP-43 WT). Notably, the AAV1-Syn-EGFP-injected mice were the same cohort as those used in Fig. 1a. (**e**) GAST muscle quantification from AAV1-Syn-EGFP- (*n* = 10 mice) and TDP-43 WT-injected mice (*n* = 7 mice). (**f**) Brain weight quantification of AAV1-Syn-EGFP- (*n* = 10 mice), ΔNLS- (*n* = 10 mice), or ΔNLS/ΔCR-injected mice (*n* = 7 mice). (**g**) Representative human TDP-43 immunostaining in spinal cords of AAV1-Syn-ΔNLS- or ΔNLS/ΔCR-injected mice showing predominantly cytosolic localization without overt inclusions. (**h**) Representative TDP-43 and pTDP-43 immunohistochemistry in spinal cords from AAV1-Syn-EGFP-, ΔNLS-, or ΔNLS/ΔCR-injected mice (*n* = 4 mice per group). ΔNLS and ΔNLS/ΔCR mice exhibited increased cytosolic TDP-43 staining, whereas pTDP-43 immunoreactivity was elevated in ΔNLS mice and reduced in ΔNLS/ΔCR mice without widespread inclusions. (**i**) Representative immunoblots and quantification of soluble and insoluble TDP-43 fractions from spinal cords (*n* = 4 mice per group). (**j**) Alternative splicing analyses of *Sort1* and *Atg4b* in spinal cords (*n* = 3 mice per group). Representative images and immunoblots were reproduced in at least three independent experiments with similar results. Data are presented as mean ± SEM. Box-and-whisker plots show the median, interquartile range and min–max values; dots indicate individual data points. Statistical analyses were performed using one-way ANOVA with Tukey’s multiple-comparison test or two-sided unpaired Student’s *t*-test. Statistical test details are provided in the Source Data file. Source data

Most disease-associated mutations in the gene encoding TDP-43 are concentrated within its LCD, which is highly amyloidogenic and can independently induce neuronal death^15^. Despite this, the specific LCD core motif responsible for modulating TDP-43 neurotoxicity remains elusive. Residues 320 to 340 within the LCD represent an evolutionarily conserved region (CR) across species, consistent with its critical role in regulating TDP-43 functional assembly, including self-interaction, liquid–liquid phase separation (LLPS) and assembly-dependent regulatory processes such as autoregulation and selective RNA processing, while also playing an important role in TDP-43 aggregation in vitro; a slightly extended sequence containing this motif has also been implicated stabilizing patient-derived TDP-43 filaments^16–23^. TDP-43 overexpression has been consistently demonstrated to trigger robust neuronal death^24–28^. To investigate whether TDP-43-induced neuronal death could be affected by CR, enhanced green fluorescent protein (EGFP) tagged human wild type (WT, full-length) TDP-43 or a CR-deleted mutant (ΔCR, full-length, lacking the CR region) was overexpressed in cultured mouse primary cortical neurons. Interestingly, deletion of CR significantly inhibited neuronal loss caused by overexpression of WT TDP-43 (Fig. 1a and Extended Data Fig. 1a,b). Given that cytoplasmic TDP-43 has been implicated as the primary driver of WT TDP-43 overexpression-induced toxicity, while excess nuclear TDP-43 may disrupt RNA metabolism but is less toxic unless mislocalized^28,29^, we further investigated the impact of CR deletion in a cytoplasm-predominant context. Using a TDP-43 mutant with an inactivated NLS (TDP-43ΔNLS), which disrupts nuclear import and promotes cytoplasmic accumulation, we observed significantly increased neurotoxicity compared to WT TDP-43 as expected (Fig. 1a and Extended Data Fig. 1a,b). To assess the contribution of CR, we generated a ΔNLS/ΔCR double mutant lacking both the NLS and CR. Importantly, the exacerbated toxicity induced by ΔNLS was also significantly suppressed by CR deletion (Fig. 1a and Extended Data Fig. 1a,b). To validate these findings in vivo, an adeno-associated virus serotype 1 (AAV1) encoding both TDP-43 (either WT or ΔNLS) and EGFP under the neuron-specific promoter, was bilaterally injected into the lumbar spinal cord of 3-month-old WT mice, resulting in EGFP expression limited to the lumbar spinal cord without affecting the thoracic spinal cord or brain as described^30^ (Fig. 1b,c). While TDP-43 WT-injected mice showed no discernible differences for up to 2 months after injection, similar to mice injected with AAV1 encoding EGFP only (Extended Data Fig. 1c–e), mice given the TDP-43ΔNLS injection displayed clear signs of motor deficits, including hindlimb clasping, foot-dragging and sporadic paralysis as early as 4 weeks after injection (Fig. 1d–g and Supplementary Videos 1 and 2). Transgenic mice overexpressing TDP-43ΔNLS are widely used to study TDP-43-associated neurodegeneration^31,32^. Thus, we chose to focus on TDP-43ΔNLS to investigate the effects of CR loss on TDP-43 neuronal toxicity in vivo. Beyond the observed motor deficits, extensive electrophysiological, biochemical and histological analysis revealed other phenotypes like neuromuscular junction dysfunction, spinal cord motor neuron loss and skeletal muscle atrophy in TDP-43ΔNLS-injected mice (Fig. 1h–j). In line with our in vitro findings, the deletion of CR was also sufficient to remarkably alleviate all these defects induced by forced overexpression of TDP-43ΔNLS (Fig. 1d–j). Noteworthily, CR deletion has no noticeable effect on brain weight, the cytosolic localization of TDP-43ΔNLS or the splicing of TDP-43 target genes, but still significantly reduces the low levels of its phosphorylated and insoluble forms (Extended Data Fig. 1f–j). These results collectively suggest that CR is critical in mediating neuronal toxicity induced by overexpressed TDP-43.

### Identification of XL20 as a CR-binding small molecule suppressing TDP-43 neurotoxicity

**Fig. 2:**
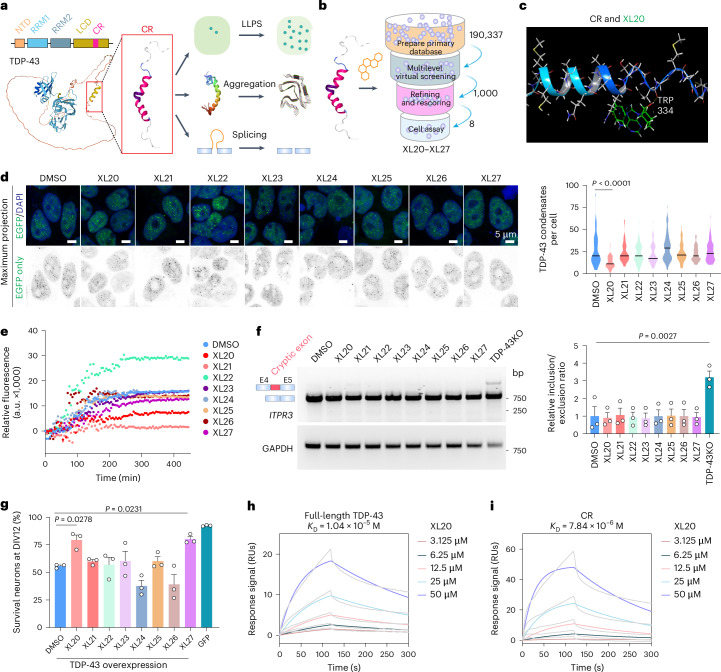
Identification of small molecules targeting the TDP-43 CR to suppress neurotoxicity. **a**, Schematic of the CR and its molecular functions. NTD, N-terminal domain. **b**, Workflow of structure-based screening for CR-targeting compounds followed by cellular validation assays. XL20–XL27 were identified as top candidates. **c**, Predicted interaction of XL20 with the CR. **d**, Representative images and quantification of TDP-43 condensates in HEK293 cells stably expressing EGFP–TDP-43 treated with dimethylsulfoxide (DMSO) or XL20–XL27 (100 µM, 18 h). Confocal images are maximum-intensity *z*-projections. XL20 exhibited significant activity at concentrations as low as 6.25 µM (Extended Data Fig. 2). More than 57 cells from 3 independent experiments were analyzed per condition. **e**, ThT fluorescence assays of recombinant TDP-43 LCD proteins incubated with DMSO or XL20–XL27 (*n* = 3 independent experiments). **f**, *ITPR3* alternative splicing analyses in HEK293 cells treated with DMSO or XL20–XL27 (100 µM, 18 h; *n* = 3 independent experiments). TDP-43 knockout (KO) cells served as positive controls for cryptic exon retention. **g**, Survival analyses of primary neurons expressing EGFP–TDP-43 WT treated with DMSO or XL20–XL27 (100 µM; *n* = 3 independent neuronal cultures). **h**,**i**, SPR sensorgrams showing XL20 binding to full-length TDP-43 (**h**) or CR peptide (**i**). RUs, response units. Sensorgrams correspond to XL20 concentrations of 3.125–50 µM. Data are presented as the mean ± s.e.m. For violin plots, center lines indicate medians; bounds indicate interquartile ranges; and widths represent data density. Statistical analyses were performed using one-way ANOVA followed by Dunnett’s multiple-comparisons test. Source data

**Extended Data Fig. 2:**
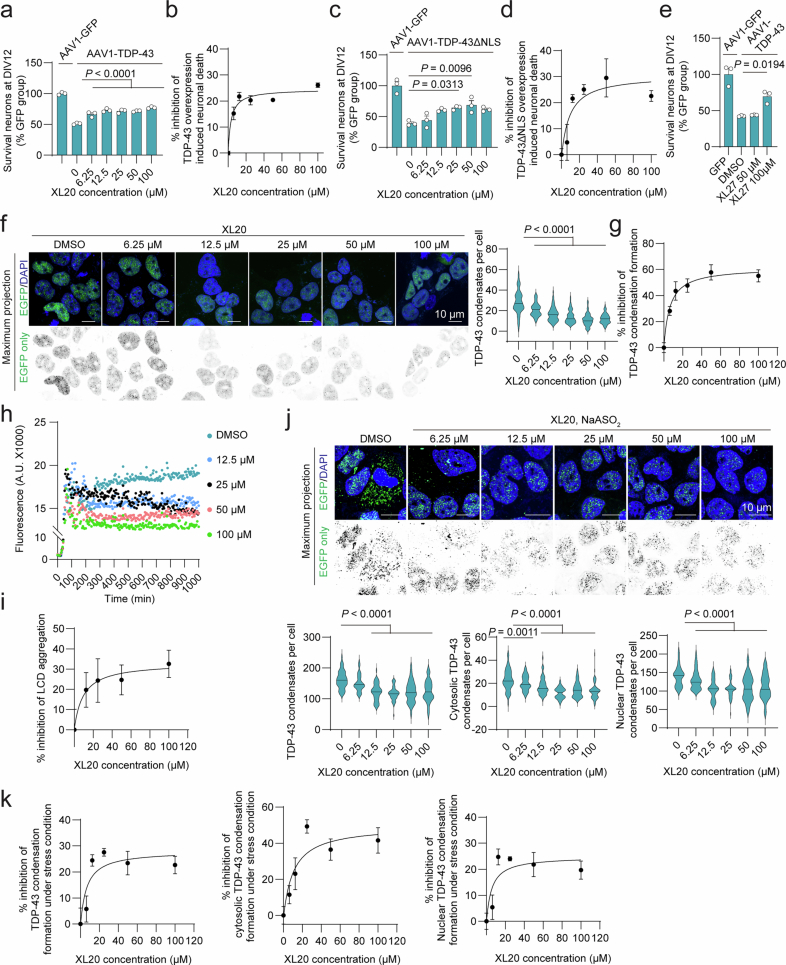
Dose-dependent effects of XL20 on TDP-43 condensation, aggregation, and neurotoxicity. (**a**–**d**) Survival and dose–response analyses of primary cortical neurons infected with AAV1-Syn-TDP-43 WT (**a**,**b**) or ΔNLS (**c**,**d**) and treated with DMSO or XL20 (6.25, 12.5, 25, 50, or 100 µM) (*n* = 3 independent neuronal cultures). (**e**) Neuroprotective effects of XL27 against TDP-43-induced neuronal death (*n* = 3 independent neuronal cultures). (**f**) Representative images and quantification of TDP-43 condensates in HEK293 cells stably expressing EGFP–TDP-43 following treatment with DMSO or XL20 (6.25–100 µM, 18 h). More than 60 cells from three independent experiments were analyzed per condition. (**g**) Dose–response curve for XL20-mediated inhibition of TDP-43 condensate formation shown in f. (**h**,**i**) ThT fluorescence assay (**h**) and dose–response quantification (**i**) of recombinant TDP-43 LCD incubated with DMSO or XL20 (12.5–100 µM) (*n* = 2–4 independent experiments). (**j**) Representative images and quantification of TDP-43 condensates in HEK293 cells stably expressing EGFP–TDP-43 following NaASO2 treatment (0.5 mM, 40 min) after pretreatment with DMSO or XL20 (6.25–100 µM, 18 h). More than 39 cells from three independent experiments were analyzed per condition. (**k**) Dose–response analyses for inhibition of stress-induced TDP-43 condensate formation shown in **j**. Representative images were reproduced in at least three independent experiments with similar results. Data are presented as mean ± SEM. For violin plots, center lines indicate medians; bounds indicate interquartile ranges; widths represent data density. Statistical analyses were performed using one-way ANOVA followed by Tukey’s multiple-comparison test. Statistical test details are provided in the Source Data file. Source data

Given the essential role of CR in mediating overexpressed TDP-43-induced neuronal toxicity, we further sought to identify pharmacological modulators that selectively engage this motif to alleviate TDP-43-related neurodegeneration in disease-relevant models (Fig. 2a). Structure-guided virtual screening of the Asinex BioDesign library (190,337 compounds) was performed to identify small molecules predicted to interact with sequence features within the CR of human TDP-43. The top 1,000 candidates ranked by docking score were further filtered for oral drug-likeness, predicted toxicophores and blood–brain barrier permeability, leading to 8 standout compounds, renamed as XL20–XL27 (Fig. 2b,c and Supplementary Fig. 1). To systematically evaluate the biological activities of XL20–XL27, we first assessed their effects on TDP-43 assembly using cell-based LLPS assays and in vitro aggregation assays as initial screening and target-engagement readouts, and then prioritized neuronal toxicity as the primary functional endpoint. Because TDP-43 assembly is closely linked to its RNA regulatory functions^18–20^, we also assessed TDP-43-dependent RNA splicing to ensure that candidate compounds did not disrupt core nuclear TDP-43 function. At the screening concentration of 100 µM, XL20, XL21 and XL23 inhibited TDP-43 LCD aggregation in vitro, but only XL20 significantly reduced endogenous nuclear liquid-like condensate formation (Fig. 2d,e). None of these compounds affected TDP-43-dependent RNA splicing, as determined by analyzing the cryptic exons in inositol 1,4,5-trisphosphate receptor type 3 (*ITPR3*) mRNA known to be regulated by TDP-43 (ref. ^33^; Fig. 2f). In primary cortical neurons overexpressing TDP-43, only XL20 demonstrated strong neuroprotection at concentrations as low as 6.25 µM, whereas XL27 required concentrations at 100 µM for a protective effect, and XL24 exacerbated neuronal death (Fig. 2g and Extended Data Fig. 2a–e). Therefore, XL20 stands out for its ability to protect neurons while also reducing condensate formation and inhibiting protein aggregation, all in a dose-dependent manner (Extended Data Fig. 2a–i). TDP-43 undergoes nuclear and cytoplasmic condensation formation in response to sodium arsenite stress, a classic inducer of canonical G3BP1/G3BP2-dependent stress granules that assemble independently of TDP-43 (ref. ^34^). We therefore quantified stress-induced TDP-43-positive condensates rather than stress granule formation per se, and found that XL20 dose dependently suppressed these TDP-43 condensates (Extended Data Fig. 2j,k).

**Extended Data Fig. 3:**
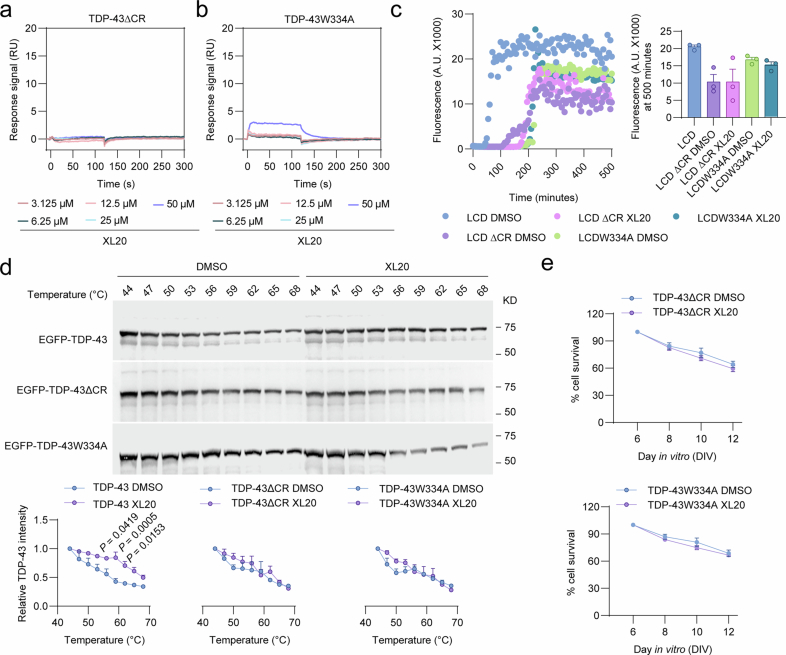
CR-dependent XL20–TDP-43 interactions regulate TDP-43 aggregation, stability, and neurotoxicity. (**a**,**b**) SPR sensorgrams showing binding of XL20 (3.125–50 µM) to immobilized TDP-43ΔCR (**a**) or TDP-43W334A (**b**). CR deletion abolished XL20 binding, whereas the W334A mutation produced weak binding responses at higher concentrations. (**c**) ThT fluorescence assays of recombinant TDP-43 LCD ΔCR and W334A proteins incubated with DMSO or XL20 (100 µM) (*n* = 3 independent experiments). Compared with WT LCD, ΔCR and W334A mutants exhibited delayed aggregation kinetics and abolished XL20-mediated suppression of fluorescence signals. (**d**) Cell lysate thermal shift assays of HEK293 cells expressing EGFP–TDP-43, EGFP–TDP-43ΔCR, or EGFP–TDP-43W334A treated with DMSO or XL20 (20 µM). Melting curves were generated from quantitative immunoblot analyses (*n* = 3 independent experiments). Representative immunoblots were reproduced in three independent experiments with similar results. (**e**) Survival analyses of primary neurons expressing EGFP–TDP-43ΔCR or W334A treated with DMSO or XL20 (20 µM) from DIV6 to DIV12 (*n* = 6 independent neuronal cultures). Data are presented as mean ± SEM. Statistical analyses were performed using two-sided unpaired Student’s *t*-test (**c**) or two-way ANOVA followed by Šídák’s or Tukey’s multiple-comparison test (**d**,**e**). Statistical test details are provided in the Source Data file. Source data

To establish direct target engagement, surface plasmon resonance (SPR) analysis was performed using recombinant full-length human TDP-43, demonstrating CR-dependent interaction of XL20 with full-length TDP-43, as deletion of the CR abolished binding (Fig. 2h and Extended Data Fig. 3a). The sensorgrams support micromolar interaction; however, deviation from a simple 1:1 model, nonlinear concentration dependence and lack of equilibrium preclude reliable kinetic analysis, and the derived affinity values are therefore interpreted as apparent estimates rather than definitive constants. CR sufficiency for XL20 interaction was further supported by SPR analysis using CR peptides alone, which showed similar weak micromolar binding (Fig. 2i). Molecular docking of the biologically active enantiomer XL20 with the (R,R,R) configuration to TDP-43 suggested a possible interaction pose at the interface of a transient α-helical segment and an adjacent extended region of the CR, incorporating hydrophobic and π–π interaction involving Trp334 (Fig. 2c). This model is presented as a structural interpretation consistent with the conformationally heterogeneous nature of the CR^21^, rather than as a definitive molecular binding mode. Consistent with this structural prediction, SPR assays showed that mutation of Trp334 (p.Trp334Ala) markedly reduced XL20 binding signal (Extended Data Fig. 3b). CR deletion suppressed TDP-43 LCD aggregation in vitro, while the p.Trp334Ala mutation reduced aggregation to a lesser extent (Extended Data Fig. 3c). In these CR-disrupted backgrounds, XL20 produced no detectable effect on TDP-43 aggregation in vitro (Extended Data Fig. 3c), consistent with CR-dependent and Trp334-dependent interaction of XL20 with TDP-43. To validate target engagement under physiologically relevant conditions, we next used an orthogonal cellular thermal shift assay^35^. XL20 significantly increased the thermal stability of WT TDP-43 in cell lysates (Extended Data Fig. 3d). In contrast, although TDP-43ΔCR and TDP-43W334A mutant exhibited modest baseline differences in thermal stability compared with WT TDP-43, XL20 did not further enhance the stability of either mutant, indicating a loss of XL20-mediated target engagement upon CR deletion or Trp334 mutation in the cellular thermal shift assay. Functionally, expression of TDP-43ΔCR or TDP-43W334A alone resulted in reduced neuronal toxicity compared with WT TDP-43, and XL20 failed to provide additional neuroprotection (Extended Data Figs. 1a and 3e), further indicating that XL20’s neuroprotective effect also requires intact CR-dependent and Trp334-dependent target engagement. Together, these data identify XL20 as a CR-engaging small molecule that selectively suppresses CR-dependent TDP-43 assembly behaviors including LLPS and aggregation, and confers potent neuroprotection at low micromolar concentrations, establishing XL20 as a lead compound for therapeutic development.

### Safety and pharmacokinetics of XL20 in mice

**Fig. 3:**
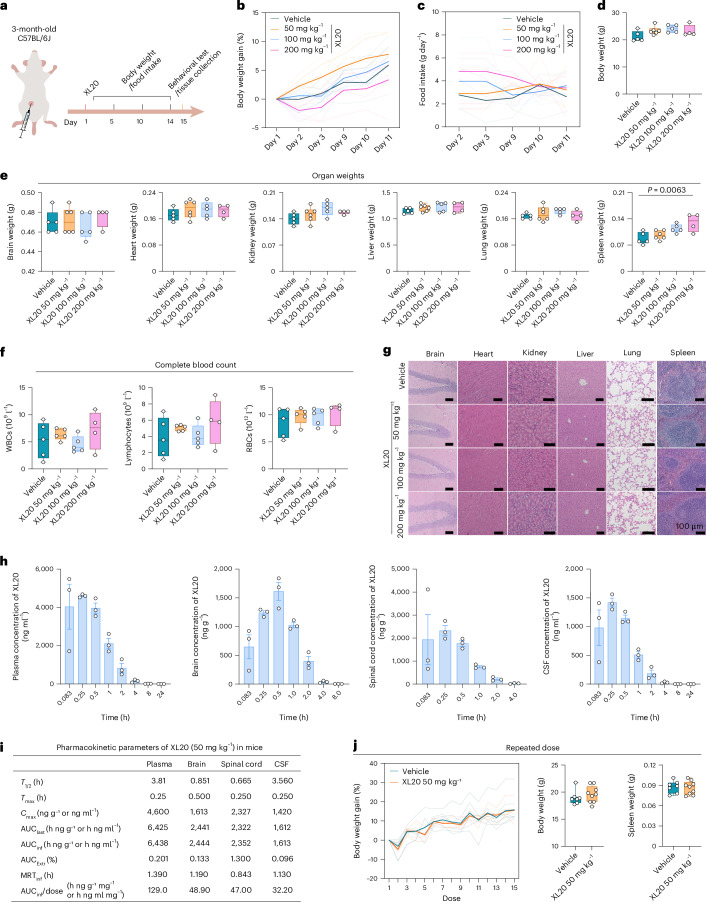
Safety and pharmacokinetic analyses of XL20 in mice. **a**, Schematic of the single-dose toxicity study. WT mice received vehicle or XL20 (50, 100 or 200 mg per kg body weight) by i.p. injection on day 1. Body weight and food intake were monitored for 14 days followed by blood and tissue collection on day 15. **b**–**f**, Body weight gain (**b**), food intake (**c**), body weight (**d**), organ weight (**e**) and complete blood count analyses (**f**) of female WT mice treated with vehicle or XL20 (*n* = 5 vehicle, 5 XL20 50 mg per kg body weight, 5 XL20 100 mg per kg body weight and 4 XL20 200 mg per kg body weight mice). In **b** and **c**, line plots show mean body weight gain and food intake over time; thin lines indicate individual mice. **g**, Representative hematoxylin and eosin staining of major organs from vehicle-treated and XL20-treated female WT mice. Representative images were reproduced in at least three independent experiments with similar results. **h**, Pharmacokinetic analyses of XL20 concentrations in plasma, CSF, brain and spinal cord collected at indicated time points following XL20 administration (50 mg per kg body weight; *n* = 3 mice per time point). **i**, Pharmacokinetic parameters calculated from the data shown in **h**. **j**, Body weight gain and body weight analyses of female WT mice treated with vehicle or XL20 for 15 repeated doses (*n* = 7 vehicle and 9 XL20 mice). Data are presented as the mean ± s.e.m. Box-and-whisker plots show the median, interquartile range and min–max values; dots indicate individual data points. Statistical analyses were performed using one-way ANOVA followed by Tukey’s multiple-comparison test or two-sided Student’s *t*-test. WBC, white blood cell; RBC, red blood cell; *T*_½_, elimination half-life; *T*_max_, time to maximum concentration; *C*_max_, maximum concentration; AUC, area under the concentration–time curve (where last denotes the last measurable concentration, inf infinity and Extr extrapolated); MRT_inf_, mean residence time to infinity. Source data

Before testing the in vivo efficacy of XL20, we sought to assess its safety and determine the appropriate dosing regimen in mice. To achieve this, we first performed a single-dose toxicology study by injecting WT mice intraperitoneally (i.p.) with varying doses of XL20, that is, 50 mg, 100 mg and 200 mg per kg body weight per dose, and monitored the behavioral and/or histopathological changes in these mice for 2 weeks (Fig. 3a). While one of five mice in the 200 mg per kg body weight group died on day 10 after injection, no animal deaths were observed in the other groups (Supplementary Fig. 2a). At the highest dose, decreased body weight gain and food intake were observed, while no obvious changes in body or organ weight were noted in the lower-dose groups (Fig. 3b–e and Supplementary Fig. 2b). Interestingly, the 50 mg per kg body weight group showed slightly, but not significantly, higher body weight gain compared to the vehicle and 100 mg per kg body weight groups (Fig. 3b). Complete blood count tests showed comparable numbers of white blood cells, red blood cells and platelets across all groups, indicating no disturbance in hematological health (Fig. 3f and Supplementary Fig. 2c). Consistently, histological examination revealed no pathological abnormalities in vital organs across all groups (Fig. 3g). To evaluate the pharmacokinetics of XL20, a dose of 50 mg per kg body weight was first administered, revealing rapid distribution with peak concentrations in plasma, spinal cord and cerebrospinal fluid (CSF) within 15 min, and in the brain within 30 min (Fig. 3h,i). The half-life of XL20 was 3.81 h in plasma, 3.56 h in CSF, 0.851 h in the brain and 0.665 h in the spinal cord (Fig. 3h,i). A lower dose of 5 mg per kg body weight achieved maximal concentrations of 223 ng ml^−^^1^ in plasma and 23 ng g^−1^ in brain tissue, while a 50 mg per kg body weight dose increased these levels to 4,600 ng ml⁻^1^ and 1,613 ng g^−1^, respectively, indicating the nonlinear pharmacokinetic behavior of XL20 likely due to saturation of clearance pathways or increased brain penetration efficiency at higher systemic exposures (Fig. 3h,i and Supplementary Fig. 2d,e). Both the brain-to-plasma and spinal-to-plasma concentration ratios averaged above 0.5 and approached 0.8, respectively, indicating that XL20 exhibits good CNS penetration (Supplementary Fig. 2f). Nevertheless, given the relatively short half-life of XL20 in the CNS, a multiple-dose toxicology study with repeated doses of 50 mg per kg body weight was further performed, involving i.p. administration in WT mice three times weekly for one month (Supplementary Fig. 2g). The results showed that this dosing regimen was safe and did not cause changes in body weight gain, unlike the 200 mg per kg body weight single-dose group (Fig. 3j). To further assess the long-term consequences of CR modulation by XL20 in vivo, we also examined our recently generated young and aged ΔCR mice with constitutive partial deletion of the CR^36^. Although young heterozygous TDP-43ΔCR mice show behavioral abnormalities linked to increased protein translation^36^, these effects normalize by adulthood, with performance comparable to WT mice (Supplementary Fig. 3a–d). Additionally, 18-month-old TDPΔCR mice showed no TDP-43 aggregation, neuron death or survival deficits but exhibited promising suppression of age-related neuroinflammation, evidenced by reduced microglial activation (Supplementary Fig. 3e–l). Therefore, together with its lack of effects on tau or amyloid-β aggregation and on fused-in-sarcoma (FUS) or heterogeneous nuclear ribonucleoprotein A1 (hnRNPA1) LLPS (Supplementary Fig. 4), XL20 is a potential CNS-penetrant compound candidate with a favorable safety profile under an appropriate dosing regimen.

### XL20 attenuates TDP-43 neurotoxicity in mice and human motor neurons

**Fig. 4:**
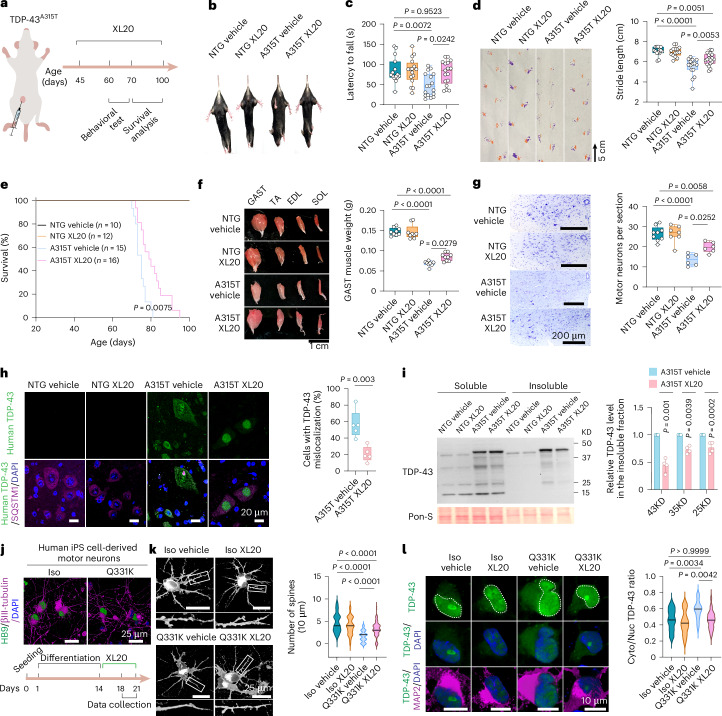
XL20 slows disease progression in mutant TDP-43 transgenic mice. **a**, Schematic of XL20 administration. **b**–**d**, Tail suspension (**b**), rotarod (**c**; *n* = 15 NTG vehicle, 16 NTG XL20, 17 p.Ala315Thr vehicle and 17 p.Ala315Thr XL20 mice) and footprint analyses with stride length quantification (**d**; *n* = 14 NTG vehicle, 15 NTG XL20, 15 p.Ala315Thr vehicle and 18 p.Ala315Thr XL20 mice) from 70-day-old NTG and p.Ala315Thr mice treated beginning at 45 days of age. **e**, Kaplan–Meier survival analyses (*n* = 10 NTG vehicle, 12 NTG XL20, 15 p.Ala315Thr vehicle and 16 p.Ala315Thr XL20 mice). **f**–**i**, GAST muscle (**f**; *n* = 9 NTG vehicle, 9 NTG XL20, 8 p.Ala315Thr vehicle and 9 p.Ala315Thr XL20 mice), motor neuron (**g**; *n* = 9 NTG vehicle, 7 NTG XL20, 6 p.Ala315Thr vehicle and 6 p.Ala315Thr XL20 mice), TDP-43 mislocalization (**h**; n = 5 mice per group) and immunoblotting analyses of insoluble TDP-43 normalized to Ponceau S staining (**i**; *n* = 4 mice per group). **j**, Immunostaining of iPS cell-derived motor neurons using HB9 and βIII-tubulin. **k**,**l**, Microtubule-associated protein 2 (MAP2) staining with dendritic spine quantification (**k**) and TDP-43 localization (**l**) in motor neurons treated with vehicle or XL20 (20 µM, 7 days). More than 59 dendrites or 25 neurons from 3 independent experiments were analyzed per group. Data are presented as the mean ± s.e.m. Box-and-whisker plots show the median, interquartile range and min–max values. Violin plots show medians, interquartile ranges and data density. One-way ANOVA with Tukey’s multiple-comparison test or two-sided unpaired Student’s *t*-test. Source data

**Extended Data Fig. 4:**
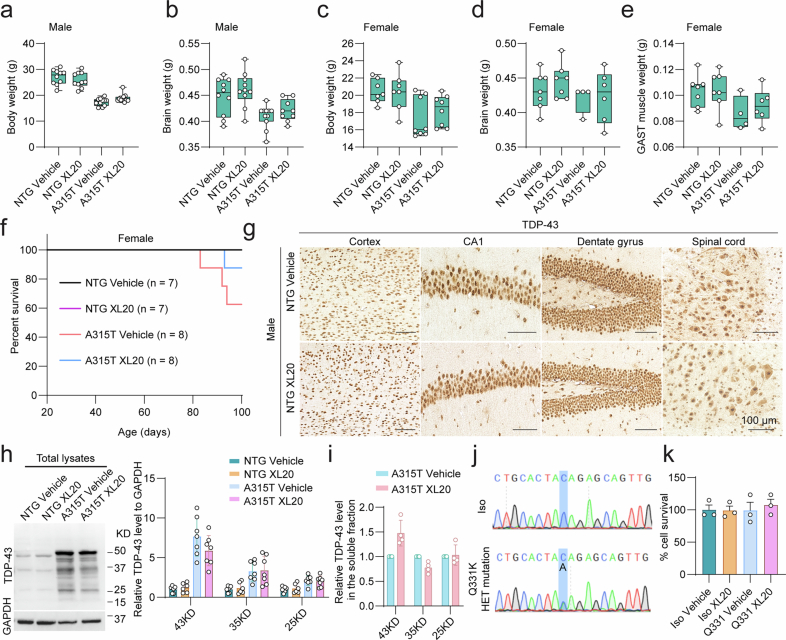
Effects of XL20 in TDP-43A315T mice and iPSC-derived motor neurons. (**a**,**b**) Body weight (**a**; *n* = 10 NTG vehicle, 10 NTG XL20, 11 A315T vehicle, and 12 A315T XL20 mice) and brain weight (**b**; *n* = 10 NTG vehicle, 10 NTG XL20, 11 A315T vehicle, and 9 A315T XL20 mice) of age-matched male NTG and end-stage male A315T mice treated with vehicle or XL20. (**c**–**e**) Body weight (**c**; *n* = 7 NTG vehicle, 7 NTG XL20, 8 A315T vehicle, and 8 A315T XL20 mice), brain weight (**d**; *n* = 7 NTG vehicle, 7 NTG XL20, 4 A315T vehicle, and 6 A315T XL20 mice), and GAST muscle weight (**e**; *n* = 7 NTG vehicle, 7 NTG XL20, 4 A315T vehicle, and 6A315T XL20 mice) of 100-day-old female NTG and A315T mice treated with vehicle or XL20. (**f**) Kaplan–Meier survival analyses (*n* = 7 NTG vehicle, 7 NTG XL20, 8 A315T vehicle, and 8 A315T XL20 mice). (**g**) Representative TDP-43 immunohistochemistry in male NTG mice treated with vehicle or XL20 (*n* = 4 mice per group). (**h**) Representative immunoblots and quantification of TDP-43 in spinal cord lysates from NTG and A315T mice treated with vehicle or XL20 (*n* = 6 NTG vehicle, 6 NTG XL20, 7 A315T vehicle, and 7 A315T XL20 mice). (**i**) Quantification of soluble TDP-43 fractions corresponding to **h** (*n* = 4 mice per group). (**j**) DNA sequencing analyses of iPSC-derived motor neurons. (**k**) Survival analyses of iPSC-derived motor neurons (20 µM XL20, 7d; *n* = 3 independent cultures). Representative images and immunoblots were reproduced in at least three independent experiments with similar results. Data are presented as mean ± SEM. Box-and-whisker plots show the median, interquartile range and min–max values; dots indicate individual data points. Statistical analyses were performed using one-way ANOVA followed by Tukey’s multiple-comparison test. Statistical test details are provided in the Source Data file. Source data

TDP-43 p.Ala315Thr hemizygous mice (p.Ala315Thr mice) on the C57BL/6J genetic background are widely used TDP-43 transgenic mouse models with robust neurodegeneration, motor abnormalities and early lethality^37–41^. We treated p.Ala315Thr mice with XL20 beginning at 45 days of age—well after the onset of fasciculations and facial spasms observed around day 22—using the identified safe dosing regimen of 50 mg per kg body weight per dose via i.p. injection three times weekly (Fig. 4a). At around 70 days of age, p.Ala315Thr mice show hindlimb clasping and reduced performance on the rotarod test, indicating impaired motor coordination (Fig. 4b,c and Supplementary Video 3). Remarkably, administration of XL20 eliminated hindlimb clasping and enhanced rotarod test performance to levels similar to those of age-matched non-transgenic littermates (NTG mice; Fig. 4b,c and Supplementary Video 3). Further gait analysis demonstrated that XL20 also significantly alleviated gait abnormalities in p.Ala315Thr mice (Fig. 4d). Importantly, in contrast to vehicle-treated male p.Ala315Thr mice, those receiving XL20 exhibited a notable increase in lifespan (Fig. 4e and Extended Data Fig. 4a,b). And, despite the considerable variability in survival, female TDP-43 p.Ala315Thr hemizygous mice treated with XL20 also exhibited the potential extension of lifespan (Extended Data Fig. 4c–f). ALS is primarily characterized by progressive loss of skeletal muscles and motor neurons. Histopathological analysis of end-stage mice revealed that XL20-treated p.Ala315Thr mice showed significantly alleviated core pathological features, despite being older and having experienced a more prolonged disease duration than vehicle-treated controls (Fig. 4f,g). Consistent with previous reports^41^, immunostaining confirmed extensive diffuse cytoplasmic TDP-43 accumulation and rare p62-positive cytoplasmic aggregates in p.Ala315Thr spinal motor neurons, whereas NTG neurons showed predominantly nuclear TDP-43 localization^42^ (Fig. 4h and Extended Data Fig. 4g). As expected, XL20 treatment effectively suppressed these pathological features, aligning with its role as a potent suppressor of LLPS, the process known to facilitate cytoplasmic TDP-43 accumulation^43^. Supporting these findings, immunoblot analysis following detergent-based cellular fractionation further revealed that XL20 significantly reduced insoluble TDP-43, particularly its cleaved C-terminal fragments, in p.Ala315Thr mice without affecting the soluble form (Fig. 4i and Extended Data Fig. 4h,i). To validate this specificity and efficacy in a physiologically relevant system, we used human induced pluripotent stem (iPS) cell-derived motor neurons bearing the heterozygous TDP-43 p.Gln331Lys mutation and their isogenic controls with the disease-relevant mutation corrected (p.Gln331Lys and Iso motor neurons obtained from iXCells Biotechnologies with certificate of analysis; Fig. 4j and Extended Data Fig. 4j). While p.Gln331Lys motor neurons showed no nuclear TDP-43 depletion, aggregation or survival differences compared to Iso controls, they exhibited reduced spine density and increased cytoplasmic TDP-43 accumulation (Fig. 4k,l). XL20 at 20 μM, a concentration selected based on dose–response studies showing near-maximal neuroprotection, reversed these abnormalities, restoring spine density and cytoplasmic TDP-43 levels to those of isogenic controls, without affecting neuronal viability (Fig. 4k,l and Extended Data Fig. 4k). These results, together with our in vivo findings, suggest that XL20 can effectively suppress cytoplasmic TDP-43 accumulation and likely associated neurotoxicity.

### Genetic deletion or pharmacological engagement of the CR enhances bioenergetics

**Fig. 5:**
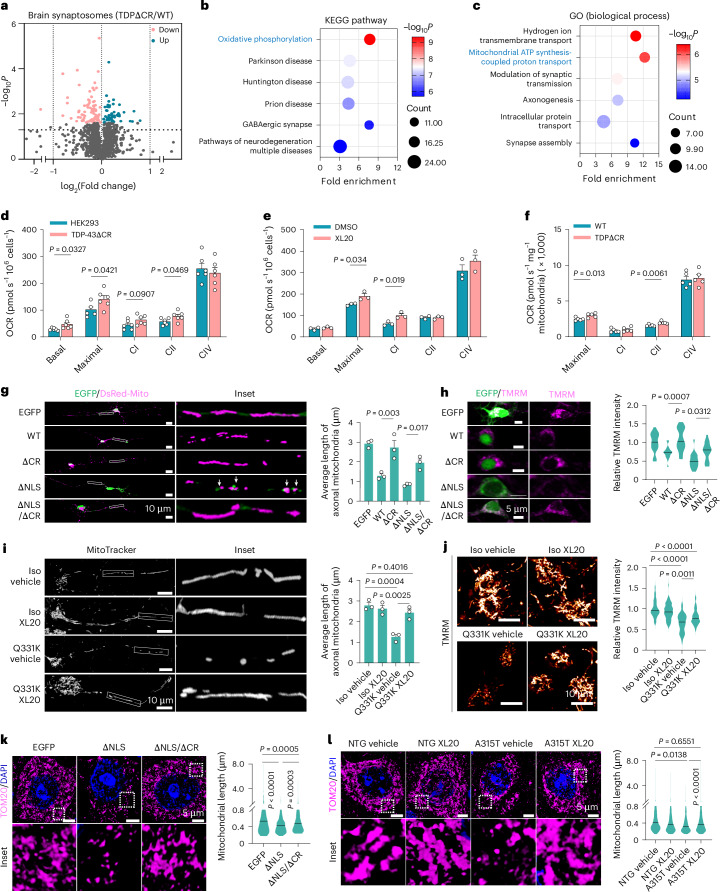
Genetic deletion or pharmacological engagement of the CR improves mitochondrial function and suppresses mitochondrial TDP-43 accumulation. **a**–**c**, Volcano plot (**a**), KEGG enrichment (**b**) and GO enrichment (**c**) analyses of differentially expressed proteins in synaptosomes (*n* = 4 mice per group). **d**–**f**, Oxygen consumption rate (OCR) analyses of HEK293ΔCR cells (**d**; *n* = 6 independent experiments), HEK293 cells treated with XL20 (**e**; 100 µM, 18 h; *n* = 3 independent experiments) and isolated mitochondria from mice (**f**; *n* = 5 mice per group). CI, CII and CIV refer to mitochondrial complexes I, II and IV, respectively. **g**,**h**, Mitochondrial morphology (**g**; *n* = 68 EGFP, 66 WT, 68 ΔCR, 68 ΔNLS and 52 ΔNLSΔCR from 3 independent experiments) and tetramethylrhodamine methyl ester (TMRM) staining (**h**; *n* = 20 EGFP, 17 WT, 20 ΔCR, 18 ΔNLS and 17 ΔNLS/ΔCR from 3 independent experiments) in primary neurons expressing DsRed-Mito. Arrowheads indicate mitochondria-associated TDP-43 condensates. **i**,**j**, Mitochondrial morphology (**i**; *n* = 69 Iso vehicle, 71 Iso XL20, 76 p.Gln331Lys vehicle and 70 p.Gln331Lys XL20 from 3 independent experiments) and TMRM intensity (**j**; *n* = 61 Iso vehicle, 66 Iso XL20, 49 p.Gln331Lys vehicle and 62 p.Gln331Lys XL20 from 3 independent experiments) in iPS cell-derived motor neurons (20 µM XL20, 7 days). **k**,**l**, Mitochondrial length in spinal cord neurons of AAV1-injected mice (**k**) and p.Ala315Thr mice (**l**; *n* = 482–1,176 mitochondria from 3 mice per group). Data are presented as the mean ± s.e.m. For violin plots, center lines indicate medians; bounds indicate interquartile ranges; and widths represent data density. Statistical analyses were performed using one-way ANOVA followed by Tukey’s multiple-comparison test or two-sided unpaired Student’s *t*-test. Source data

**Extended Data Fig. 5:**
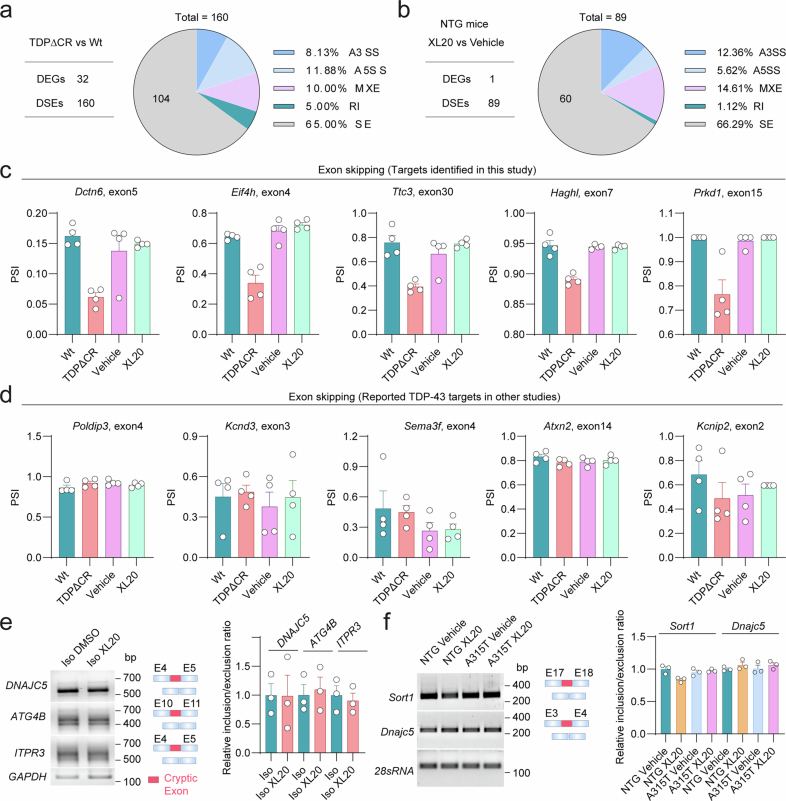
XL20 does not alter TDP-43-mediated alternative splicing. (**a**,**b**) Classification of differential alternative splicing events identified in 18-month-old TDPΔCR mice (**a**) and XL20-treated mice (**b**; 15 doses over 1 month) using thresholds of *P* < 0.05 and false discovery rate (FDR) < 0.01 (*n* = 4 mice per group). AS3SS, alternative 3′ splice site; AS5SS, alternative 5′ splice site; MXE, mutually exclusive exon; RI, retained intron; SE, skipped exon. (**c**) Percent spliced in (PSI) analyses of the top five exon-skipping events identified in TDPΔCR mice. Corresponding PSI values from vehicle- and XL20-treated mice are shown (*n* = 4 mice per group). (**d**) PSI analyses of previously reported TDP-43 splicing targets in 18-month-old TDPΔCR and XL20-treated mice (*n* = 4 mice per group; XL20, 15 doses over 1 month). (**e**,**f**) Alternative splicing analyses of reported TDP-43 targets in iPSC-derived motor neurons treated with DMSO or XL20 (20 µM, 7d) (**e**) or NTG/A315T mice treated with XL20 (**f**) (*n* = 3 independent cultures or mice per group). Representative gel images were reproduced in at least three independent experiments with similar results. Data are presented as mean ± SEM. Statistical analyses were performed using one-way ANOVA followed by Tukey’s multiple-comparison test or two-sided unpaired Student’s *t*-test. Statistical test details are provided in the Source Data file. Source data

**Extended Data Fig. 6:**
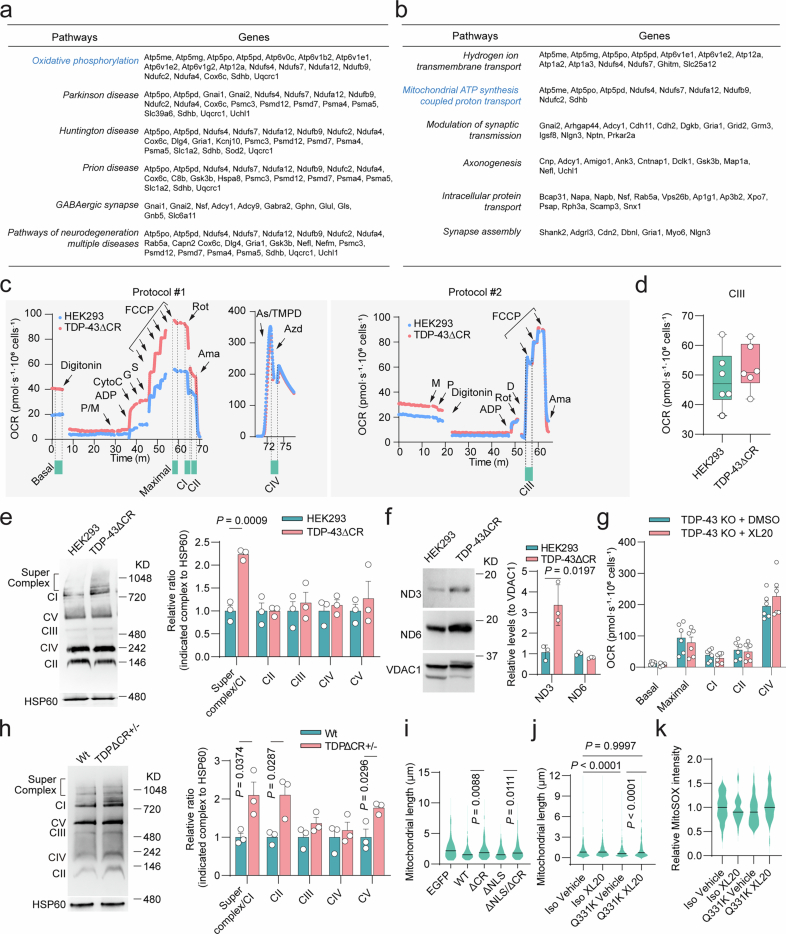
CR deletion enhances mitochondrial respiration. (**a**,**b**) Differentially expressed synaptosome proteins across multiple GO pathways. (**c**) Oxygen consumption rate (OCR) analyses of HEK293 cells using protocol #1 (Complex I, II, and IV activities) and protocol #2 (Complex III activity). P, pyruvate; M, malate; CytoC, cytochrome c; G, glutamate; S, succinate; Rot, rotenone; Ama, antimycin A; D, tetramethylhydroquinone. (**d**) Quantification of Complex III activity in HEK293 cells (*n* = 6 independent experiments). (**e**) Representative immunoblots and quantification of OXPHOS complex assembly in mitochondrial fractions from HEK293 cells (*n* = 3 independent experiments). (**f**) Representative immunoblots and quantification of ND3 and ND6 expression in HEK293 cells (*n* = 3 independent experiments). (**g**) OCR analyses of TDP-43 knockout and TDP-43ΔCR HEK293 cells treated with vehicle or XL20 (100 µM, 18 h; *n* = 6 independent experiments). (**h**) Representative immunoblots and quantification of OXPHOS complex assembly in mitochondrial fractions from WT and hemizygous TDPΔCR mice at 5 months of age (*n* = 3 mice per group). (**i**,**j**) Quantification of mitochondrial length in dendrites and axons of primary neurons expressing EGFP, EGFP–TDP-43 WT, ΔCR, ΔNLS, or ΔNLS/ΔCR (**i**), and in isogenic or heterozygous TDP-43 Q331K iPSC-derived motor neurons treated with vehicle or XL20 (20 µM) (**j**). More than 200 mitochondria per group were analyzed across three independent experiments. (**k**) Mitochondrial ROS measurements in iPSC-derived motor neurons treated with vehicle or XL20 (20 µM, 7d; *n* = 3 independent cultures). Representative immunoblots were reproduced in at least three independent experiments with similar results. Data are presented as mean ± SEM. For violin plots, center lines indicate medians; bounds indicate interquartile ranges; widths represent data density. Statistical analyses were performed using one-way ANOVA followed by Tukey’s multiple-comparison test or two-sided unpaired Student’s *t*-test. Statistical test details are provided in the Source Data file. Source data

TDP-43 overexpression triggers neuronal death without prominent protein aggregation, as seen in TDP-43ΔNLS and p.Ala315Thr mouse models, while p.Gln331Lys motor neurons show no TDP-43 aggregation, together suggesting that CR modulation should not confer neuroprotection through protein aggregation-dependent mechanisms. Additionally, there is no widespread nuclear depletion of endogenous TDP-43 in these models. Consistently, no significant changes in many previously identified TDP-43 RNA targets were observed in young and aged ΔCR mice, p.Ala315Thr mice and human p.Gln331Lys motor neurons alone, or WT mice, p.Ala315Thr mice and human p.Gln331Lys motor neurons treated with XL20 (Extended Data Fig. 5). Together with the findings that XL20 does not affect TDP-43 RNA splicing activity and, notably, that CR deletion could abolish neuronal death induced by cytoplasmic localized TDP-43ΔNLS, these results further indicate that CR modulation should not primarily act through a splicing-dependent, nuclear mechanism to protect neurons either. Very few differentially expressed genes (DEGs) were identified in RNA-sequencing analysis, and these DEGs did not show enrichment for any known biological pathways. Synaptic loss is a prominent and early pathological feature of TDP-43-associated neurodegenerative diseases^44^. To explore neuron-specific pathways affected by CR modulation, we performed quantitative mass spectrometry analysis of synaptosomes isolated from the brains of either WT or heterozygous TDPΔCR mice. In total, 236 differentially expressed proteins were identified in TDPΔCR mice, with 150 downregulated and 86 upregulated (Fig. 5a). Intriguingly, both Kyoto Encyclopedia of Genes and Genomes (KEGG) and Gene Ontology (GO) enrichment analyses revealed that differentially expressed proteins in TDPΔCR synaptosomes were predominantly involved in mitochondrial bioenergetics (Fig. 5b,c and Extended Data Fig. 6a,b).

To gain insight into these proteomic findings, we evaluated mitochondrial respiration in HEK293 cells expressing endogenous human TDP-43 without CR (TDPΔCR cells)^36^. TDPΔCR cells showed significantly increased basal and maximal respiratory capacities in the electron transfer system, with particular enhancements in the capacities for complex I and II compared to WT cells; however, complex III and IV capacity remained unchanged (Fig. 5d and Extended Data Fig. 6c,d). Similarly, WT HEK293 cells treated with XL20 also demonstrated elevated respiration rates for maximal and complex I, when compared to those of vehicle-treated cells (Fig. 5e). Consistently, the assembly of complex I or its supercomplex was greatly enhanced (Extended Data Fig. 6e). The upregulation of complex I subunits ND3/6 in response to TDP-43 CR deletion was also noted (Extended Data Fig. 6f). Importantly, XL20 failed to enhance mitochondrial bioenergetics in either TDP-43 knockout or TDPΔCR cells (Extended Data Fig. 6g). In vivo validation using mitochondria from TDPΔCR mouse brains similarly demonstrated elevated respiratory capacity and enhanced assembly of complex I and its supercomplex, accompanied by additional increases in complex II and V assembly (Fig. 5f and Extended Data Fig. 6h). Beyond respiratory capacity and electron transport chain assembly, we next examined whether CR engagement also modulates other aspects of mitochondrial function. In cultured neurons, expression of WT TDP-43 disrupted mitochondrial morphology and membrane potential, effects that were intensified by cytoplasmic mislocalization through ΔNLS and markedly reversed by removal of the CR (Fig. 5g,h and Extended Data Fig. 6i). In p.Gln331Lys iPS cell-derived human motor neurons, XL20 treatment greatly restored mitochondrial length and membrane potential without affecting reactive oxygen species production (Fig. 5i,j and Extended Data Fig. 6j,k). Similar improvements in mitochondrial morphology were observed in vivo, as mitochondrial fragmentation in both TDP-43ΔNLS-expressing mice and p.Ala315Thr transgenic mice was reduced by either genetic CR deletion or pharmacological treatment with XL20 (Fig. 5k,l). These findings imply that mitochondrial bioenergetics may serve as a downstream target of CR engagement.

### Genetic deletion or pharmacological engagement of the CR suppresses mitochondria-associated TDP-43 likely via LLPS

**Fig. 6:**
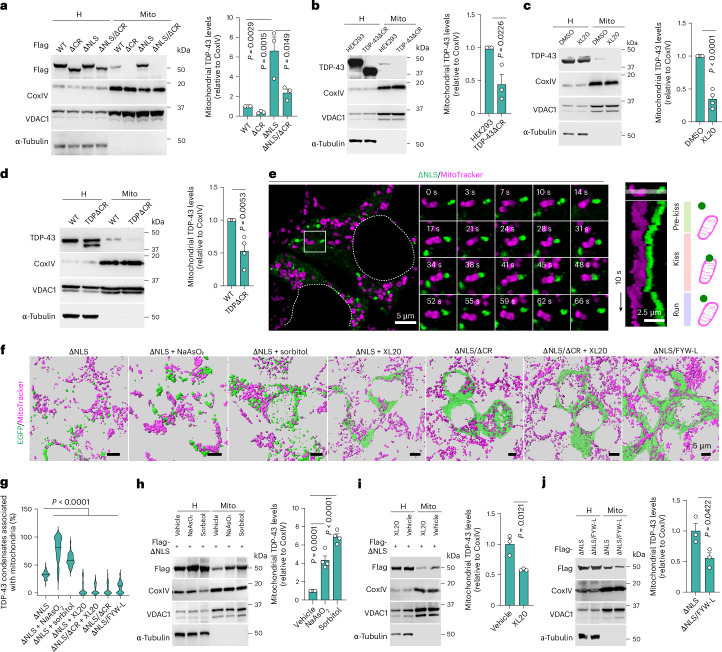
Genetic deletion or pharmacological engagement of the CR regulates TDP-43 mitochondrial localization through LLPS. **a**–**d**, Immunoblot and quantification of mitochondrial TDP-43 in HEK293 cells expressing TDP-43 WT, ΔCR, ΔNLS or ΔNLS/ΔCR (**a**; *n* = 3 independent experiments), HEK293 and HEK293ΔCR cells (**b**; *n* = 3 independent experiments), HEK293 cells treated with vehicle or XL20 (**c**; 100 µM, 18 h; *n* = 4 independent experiments) and WT and TDPΔCR mice (**d**; *n* = 4 mice per group). **e**, Representative time-lapse imaging of TDP-43 condensates (green) and mitochondria (purple) in cells expressing EGFP–TDP-43ΔNLS. Enlarged regions are shown on the right. Dashed circles indicate nuclei. **f**,**g**, Representative three-dimensional (3D) images (**f**) and quantification (**g**) of mitochondria-associated TDP-43 condensates in cells expressing EGFP–TDP-43ΔNLS, EGFP–TDP-43ΔNLS/ΔCR or EGFP–TDP-43ΔNLS/FYW-L treated with NaAsO_2_ (0.5 mM), sorbitol (0.5 M) and/or XL20 (100 µM, 18 h). For **g**, *n* = 52 ΔNLS, 42 ΔNLS + NaAsO_2_, 45 ΔNLS + sorbitol, 37 ΔNLS/ΔCR, 45 ΔNLS/FYW-L, 45 ΔNLS + XL20 and 47 ΔNLS/ΔCR + XL20 cells from 3 independent experiments, including cells with zero detectable mitochondria-associated condensates. **h**–**j**, Immunoblot and quantification of mitochondrial TDP-43 in indicated cells exposed to NaAsO_2_ or sorbitol (**h**; *n* = 4 independent experiments), treated with XL20 (**i**; *n* = 3 independent experiments) or expressing EGFP–TDP-43ΔNLS or EGFP–TDP-43ΔNLS/FYW-L (**j**; *n* = 3 independent experiments). Representative images and immunoblots were reproduced in at least three independent experiments with similar results. Data are presented as the mean ± s.e.m. Statistical analyses were performed using one-way ANOVA followed by Tukey’s multiple-comparison test or two-sided unpaired Student’s *t*-test. Source data

A substantial and ever-expanding body of research has revealed mitochondria-associated TDP-43 in provoking mitochondrial dysfunction in neurodegenerative diseases^45–51^. To delineate how mitochondrial bioenergetics is affected by CR, we next determined whether TDP-43 mitochondrial localization was perturbed by CR deletion or XL20. Subcellular fractionation and immunoblot of exogenously expressed flagged TDP-43 in HEK293 cells revealed that TDP-43ΔNLS showed enhanced mitochondrial localization compared to WT TDP-43, while the CR deletion abolished mitochondrial localization for both WT TDP-43 and TDP-43ΔNLS (Fig. 6a). We confirmed the significant inhibitory effect of CR deletion and XL20 on endogenous TDP-43 levels in the mitochondria fraction in mice or HEK293 cells even though their basal levels were low as reported^51,52^ (Fig. 6b–d). Noteworthily, mitochondrial markers CoxIV and VDAC1 remained unchanged with CR deletion and XL20 treatment, consistent with previous studies indicating that mitochondrial TDP-43 does not influence mitochondrial biogenesis^51,53^. Additionally, in heterozygous TDPΔCR mice, the upper immunoblot band representing WT endogenous TDP-43 also decreased in the mitochondrial fraction (Fig. 6d).

**Extended Data Fig. 7:**
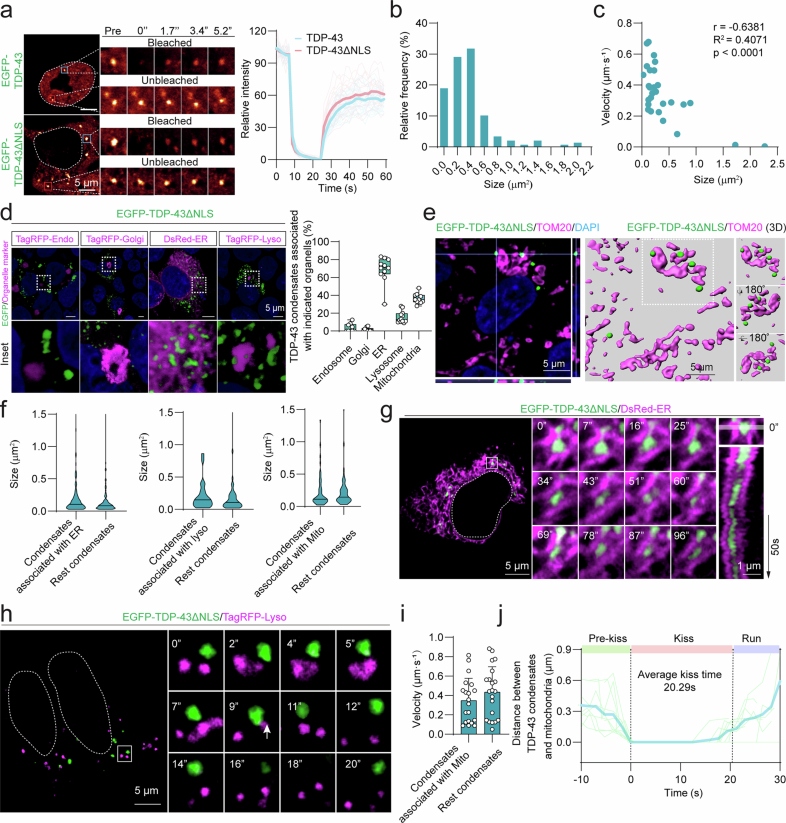
TDP-43 condensates dynamically associate with mitochondria. (**a**) FRAP analyses of TDP-43 condensates in HEK293 cells expressing EGFP–TDP-43 or EGFP–TDP-43ΔNLS. A total of 19 EGFP–TDP-43 condensates and 25 EGFP–TDP-43ΔNLS condensates from three independent experiments were analyzed. Enlarged time-lapse images and normalized fluorescence recovery curves are shown. Dashed circles indicate nuclei. (**b**) Size distribution of cytosolic TDP-43 condensates. (**c**) Correlation between condensate size and movement velocity. (**d**) Association of TDP-43 condensates (green) with organelles (purple) in HEK293 cells co-expressing EGFP–TDP-43ΔNLS and organelle markers (*n* = 9 endosome, 10 Golgi, 11 ER, 12 lysosome, and 10 mitochondria cells from three independent experiments). Approximately 68% of condensates associated with the endoplasmic reticulum (ER), whereas ~15% associated with lysosomes. (**e**) Representative confocal and 3D reconstructed images of TDP-43 condensates and TOM20-positive mitochondria. (**f**) Quantification of condensate size revealed no differences between organelle-associated and non-associated condensates (>50 condensates analyzed per group from three independent experiments). (**g**) Representative time-lapse images and kymographs of TDP-43 condensates and ER showing stable interactions without prominent kiss-and-run behavior. (**h**) Representative time-lapse images of TDP-43 condensates and lysosomes showing transient interactions. Arrowheads indicate association events. (**i**) Quantification of condensate velocity showed no significant differences between mitochondria-associated and non-associated condensates. (**j**) Quantification of interaction durations between TDP-43 condensates and mitochondria (*n* = 10 interaction events from three independent experiments). Representative images were reproduced in at least three independent experiments with similar results. Data are presented as mean ± SEM. For violin plots, center lines indicate medians; bounds indicate interquartile ranges; widths represent data density. Statistical analyses were performed using two-sided unpaired Student’s *t*-tests. Statistical test details are provided in the Source Data file. Source data

**Extended Data Fig. 8:**
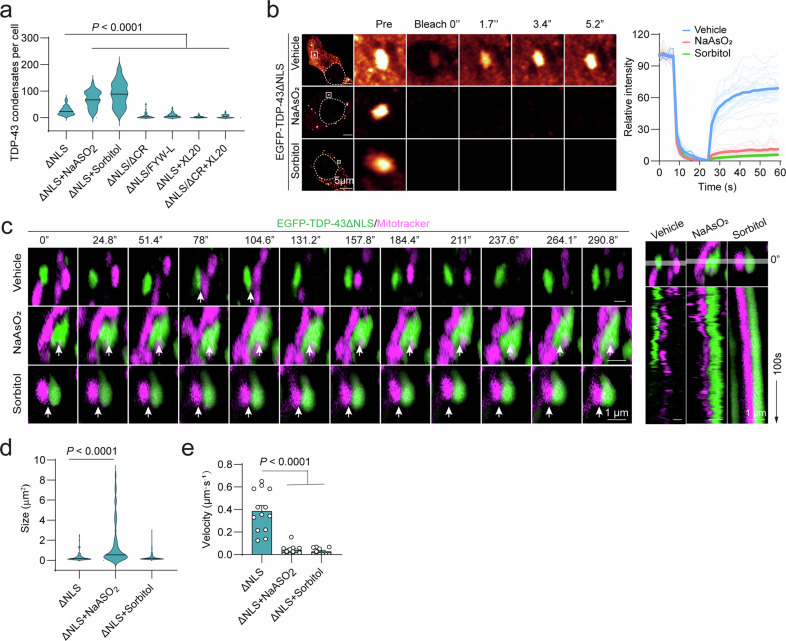
Stress enhances the association between TDP-43 condensates and mitochondria. (**a**) Quantification of TDP-43 condensates in HEK293 cells expressing EGFP–TDP-43ΔNLS treated with NaAsO_2_ (0.5 mM), sorbitol (0.5 M), or XL20 (100 µM, 18 h), or expressing EGFP–TDP-43ΔNLS/ΔCR or ΔNLS/FYW-L mutants. A total of 42-75 cells from three independent experiments were analyzed per condition. (**b**) FRAP analyses of TDP-43 condensates in EGFP–TDP-43ΔNLS-expressing cells treated with vehicle, NaAsO_2_, or sorbitol for 2 h. A total of 23 vehicle-treated, 21 NaAsO_2_-treated, and 20 sorbitol-treated condensates from three independent experiments were analyzed. Enlarged time-lapse images and normalized fluorescence recovery curves are shown. Dashed circles indicate nuclei. (**c**) Representative time-lapse images and kymographs of TDP-43 condensates (green) and mitochondria (purple) in EGFP–TDP-43ΔNLS-expressing cells treated with vehicle, NaAsO_2_, or sorbitol for 2 h. Arrowheads indicate condensate–mitochondria ‘kissing’ events. (**d**,**e**) Quantification of condensate size (**d**; *n* = 50–213 condensates from three indenpendent experiments) and motility (**e**; *n* = 10–13 condensates from three indenpendent experiments) in EGFP–TDP-43ΔNLS-expressing cells following stress treatment. NaAsO_2_ increased condensate size, whereas both NaAsO_2_ and sorbitol reduced condensate motility. Representative images were reproduced in at least three independent experiments with similar results. Data are presented as mean ± SEM. For violin plots, center lines indicate medians; bounds indicate interquartile ranges; widths represent data density. Statistical analyses were performed using one-way ANOVA followed by Tukey’s multiple-comparison test. Statistical test details are provided in the Source Data file. Source data

Since the CR is critical for both TDP-43 LLPS and mitochondrial localization, the interplay between these processes is an intriguing yet unexplored area of interest. Evaluating TDP-43 LLPS and mitochondrial association in vitro is challenging due to harsh conditions like high protein and salt concentrations. To overcome this, we expressed Flag-tagged or GFP-tagged human TDP-43 ΔNLS in HEK293 cells as described^43^, allowing us to study cytoplasmic TDP-43 LLPS behaviors alongside mitochondria in intact cellular environments. TDP-43ΔNLS formed mobile cytoplasmic droplet-like accumulations with similar dynamic properties to those of WT TDP-43 in the nucleus (Extended Data Fig. 7a). Over 80% of cytosolic TDP-43ΔNLS condensates measured less than 0.5 µm^2^, with smaller condensates exhibiting higher movement speeds (Extended Data Fig. 7b,c). These condensates colocalized with mitochondria, endoplasmic reticulum and lysosomes but not with the Golgi apparatus or endosomes (Extended Data Fig. 7d,e). Size analysis showed no significant differences between organelle-associated and non-organelle-associated condensates (Extended Data Fig. 7f). Live-cell imaging revealed that these condensates constantly associated and co-migrated with the endoplasmic reticulum, while their interactions with lysosomes were transient, lasting only a few seconds (Extended Data Fig. 7g,h and Supplementary Videos 4 and 5). Yet, these condensates showed a transient ‘kiss-and-run’ interaction only predominantly with mitochondria, with an average contact duration of 20.29 s, while their velocity showed no significant difference between mitochondria-associated and non-associated condensates (Fig. 6e, Extended Data Fig. 7i,j and Supplementary Video 6). As expected, CR deletion or XL20 treatment significantly inhibited TDP-43 condensate formation and their association with mitochondria (Fig. 6f,g and Extended Data Fig. 8a). Noteworthily, the TDP-43 condensate and mitochondria association could also be greatly suppressed by the FYW-L mutant, an LLPS-deficient mutant operating independently of the CR domain^18,33,54^ (Fig. 6f,g). Immunoblot analysis confirmed that XL20 and FYW-L treatments, like CR deletion, significantly reduced TDP-43 levels in mitochondria (Fig. 6h–j). Noteworthily, condensate–mitochondria interactions are not unique to TDP-43; proteins like UBQLN2, SQSTM1/p62 and FUS, other commonly studied neurodegeneration proteins, also exhibit similar dynamic interactions with mitochondria (Supplementary Fig. 5).

**Extended Data Fig. 9:**
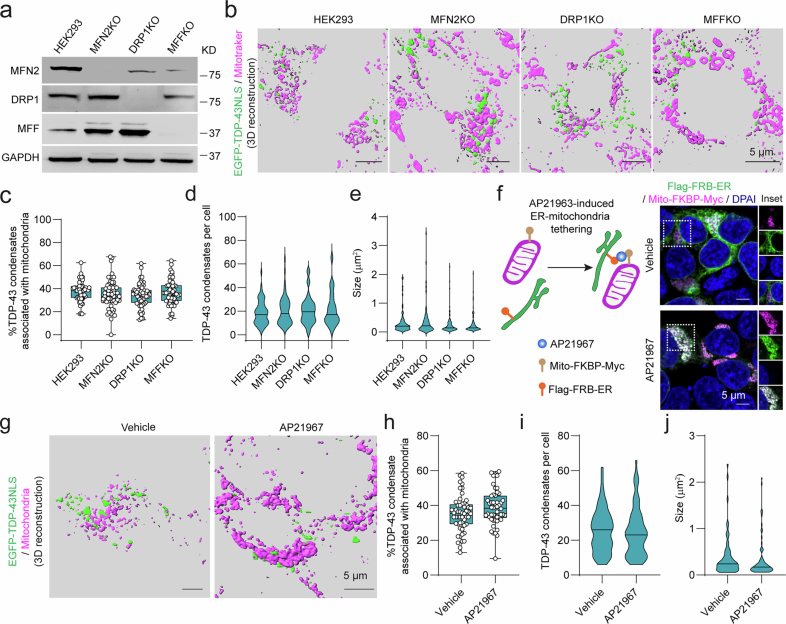
Mitochondrial dynamics and ER–mitochondria tethering do not alter TDP-43 condensate formation or mitochondrial association. (**a**) Representative immunoblots of MFN2, DRP1, and MFF in WT, MFN2KO, DRP1KO, and MFFKO HEK293 cells. (**b**) Representative 3D reconstructed images of mitochondria-associated TDP-43 condensates in EGFP–TDP-43ΔNLS-expressing HEK293, MFN2KO, DRP1KO, and MFFKO cells. (**c**–**e**) Quantification of mitochondria-associated condensates (**c**), total condensate number per cell (**d**), and condensate size (**e**) from b. More than 50 cells from three independent experiments were analyzed per group. (**f**) Schematic of AP21967-induced ER–mitochondria tethering. Representative images show ER–mitochondria association in HEK293 cells co-expressing Flag-FRBT2058L-ER and Mito-FKBP-Myc treated with vehicle or AP21967 (200 nM). (**g**) Representative 3D reconstructed images of mitochondria-associated TDP-43 condensates in EGFP–TDP-43ΔNLS-, Flag-FRBT2058L-ER-, and Mito-FKBP-Myc-expressing HEK293 cells treated with vehicle or AP21967 (200 nM). (**h**–**j**) Quantification of mitochondria-associated condensates (**h**), total condensate number per cell (**i**), and condensate size (**j**) from **g**. More than 50 cells from three independent experiments were analyzed per group. Representative images and immunoblots were reproduced in at least three independent experiments with similar results. Data are presented as mean ± SEM. Box-and-whisker plots show the median, interquartile range and min–max values; dots indicate individual data points. For violin plots, center lines indicate medians; bounds indicate interquartile ranges; widths represent data density. Statistical analyses were performed using one-way ANOVA followed by Tukey’s multiple-comparison test or two-sided unpaired Student’s *t*-test. Statistical test details are provided in the Source Data file. Source data

**Extended Data Fig. 10:**
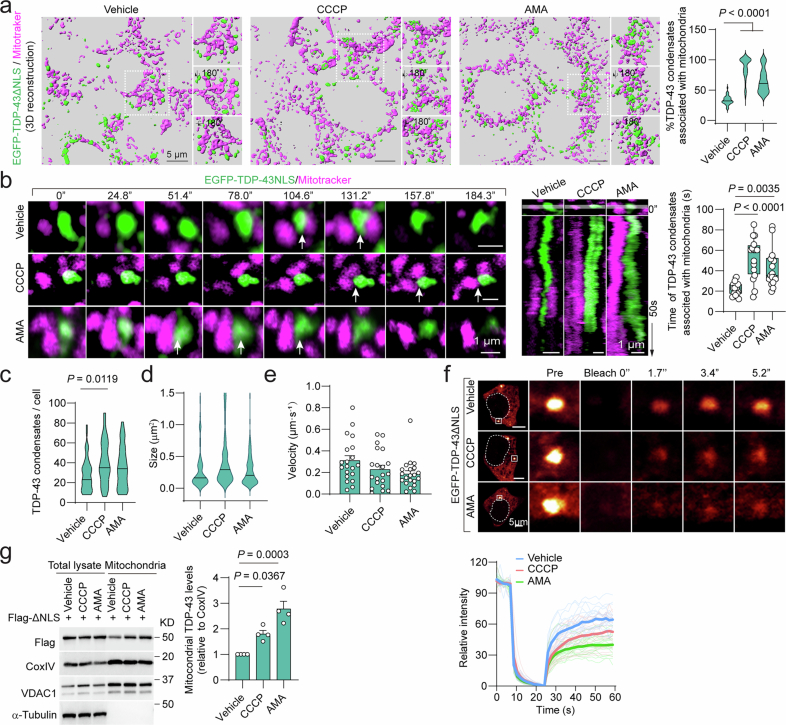
Mitochondrial stress alters TDP-43 condensate dynamics and mitochondrial association. (**a**) Representative 3D reconstructed images and quantification of TDP-43 condensates (green) associated with mitochondria (purple) in EGFP–TDP-43ΔNLS-expressing cells treated with vehicle, CCCP (20 µM), or antimycin A (AMA; 10 µM) for 2 h. A total of 38 cells from three independent experiments were analyzed per group. (**b**) Representative time-lapse images, kymographs, and quantification of condensate–mitochondria interactions in EGFP–TDP-43ΔNLS-expressing cells treated with vehicle, CCCP, or AMA for 2 h. Arrowheads indicate ‘kissing’ events. A total of 15 vehicle-treated, 16 CCCP-treated, and 17 AMA-treated interaction events from three independent experiments were analyzed. (**c**–**e**) Quantification of condensate number (**c**; *n* = 43–47 cells from three independent experiments), size (**d**; *n* = 61–102 cells from three independent experiments), and velocity (**e**; *n* = 20–21 condensates from three independent experiments) from **b**. (**f**) FRAP analyses of TDP-43 condensates in EGFP–TDP-43ΔNLS-expressing cells treated with vehicle, CCCP, or AMA for 2 h. A total of 18 vehicle-treated, 20 CCCP-treated, and 18 AMA-treated condensates from three independent experiments were analyzed. Enlarged time-lapse images and normalized fluorescence recovery curves are shown. Dashed circles indicate nuclei. (**g**) Representative immunoblots and quantification of mitochondrial TDP-43 levels in Flag-TDP-43ΔNLS-expressing HEK293 cells treated with vehicle, CCCP, or AMA for 2 h (*n* = 4 independent experiments). Representative images and immunoblots were reproduced in at least three independent experiments with similar results. Data are presented as mean ± SEM. For violin plots, center lines indicate medians; bounds indicate interquartile ranges; widths represent data density. Box-and-whisker plots show the median, interquartile range and min–max values; dots indicate individual data points.Statistical analyses were performed using one-way ANOVA followed by Tukey’s multiple-comparison test. Statistical test details are provided in the Source Data file. Source data

To determine the fate of the dynamic behavior between TDP-43 condensates and mitochondria, we examined their association under sodium arsenite and sorbitol stresses, both of which are known to induce LLPS of TDP-43 into immobilized condensates^55^. Although the size of TDP-43 condensates varied under different stress conditions, they exhibited increased numbers, reduced mobility and no photobleaching recovery, with a strikingly static, intensified association with mitochondria and elevated mitochondrial TDP-43 levels (Fig. 6f–j and Extended Data Fig. 8b–e). We next asked if mitochondria would affect the formation of cytoplasmic TDP-43 condensation by manipulating mitochondrial dynamics, endoplasmic reticulum–mitochondria tethering, or bioenergetics, various mitochondrial pathways implicated in TDP-43-assocated neurodegeneration^45–51^. In HEK293 cells lacking crucial fission and fusion regulators^56^ or with induced endoplasmic reticulum–mitochondria tethering as we described^30^, TDP-43ΔNLS expression resulted in cytoplasmic TDP-43 condensates with numbers, sizes, liquid characteristics and mitochondrial association all similar to control cells (Extended Data Fig. 9). By contrast, there was a significant rise in the formation of cytoplasmic TDP-43 condensates, paralleled by a marked increase in TDP-43 condensate and mitochondria association in response to mitochondrial uncoupler carbonyl cyanide-m-chlorophenylhydrazone (CCCP) or mitochondrial oxidative phosphorylation (OXPHOS) complex III inhibitor antimycin A (AMA) treatment (Extended Data Fig. 10a). Of note, impaired bioenergetics led to cytoplasmic TDP-43 condensates maintaining prolonged contact with mitochondria, averaging over 38 s (Extended Data Fig. 10b). Unlike sodium arsenite-induced or sorbitol-induced condensates, CCCP-induced or AMA-induced condensates remained dynamic, with slightly reduced mobility and slower fluorescence recovery after photobleaching (FRAP) but no size differences (Extended Data Fig. 10c–f). Concurrently, a significant increase in mitochondria-associated TDP-43 levels was observed after CCCP and AMA treatment (Extended Data Fig. 10g). Therefore, mitochondrial bioenergetics also play a crucial role in the formation of TDP-43 condensates, which appears to be essential for the proper localization of TDP-43 to mitochondria.

## Discussion

TDP-43 is implicated in neuronal loss across various major neurodegenerative conditions. Our study reveals the critical role of the C-terminal CR of TDP-43 in mediating its neurotoxicity, identifying this domain as a therapeutically relevant site. This work also identified XL20, a small molecule that engages this region and significantly alleviates TDP-43-induced neuronal toxicity in disease models. Given the conformationally dynamic nature of the CR^16,57–59^, we interpret XL20 binding as engagement of a locally populated CR conformational ensemble rather than interaction with a single rigid structural state. Notably, genetic deletion of the CR or its pharmacological engagement by XL20 confers neuroprotection in the absence of widespread protein aggregation, nuclear depletion or altered splicing of several TDP-43 RNA targets. Although nuclear depletion of TDP-43 has increasingly been viewed as a pathological hallmark in patient tissues, neurotoxicity can arise before, or even in the absence of, nuclear clearance in experimental models^28,29^. These findings expand our understanding of TDP-43 neurotoxicity and provide a strong foundation for the development of therapeutic strategies targeting TDP-43-associated neurodegeneration.

In our current research, we explored the role of CR in regulating TDP-43-induced neuronal toxicity. In vitro and in vivo studies consistently observed that the deletion of the CR mitigated neuronal loss caused by TDP-43. One of the key functions of TDP-43 is RNA splicing. However, young and aged heterozygous ΔCR mice show no signs of TDP-43 nuclear depletion or changes in the alternative splicing of several previously identified TDP-43 targets^36^. These findings suggest that the partial perturbation of the CR can be tolerated without disrupting several physiological functions examined here. At the same time, caution is warranted, as this region mediates TDP-43 assembly linked not only to pathological activities but also to physiological functions such as autoregulation, RNA processing and nuclear retention^16–20^. Thus, therapeutic strategies are more likely to require partial modulation of CR rather than complete disruption of its function. Along this line, we want to clarify that behavioral abnormalities previously observed in young heterozygous TDP-43ΔCR mice normalize with age, and aged TDPΔCR mice do not exhibit overt neurodegenerative phenotypes. Given that TDP-43 pathology is generally associated with impaired protein translation^60^, the increase in protein translation observed with partial CR deletion should provide benefits under such conditions. These in vivo findings are also consistent with our pharmacological approach, where XL20 likely acts as a partial modulator to attenuate CR-dependent pathological processes while preserving essential physiological functions. Noteworthily, the ability of the CR or TDP-43 to undergo LLPS is not strictly required for maintaining splicing activity for some canonical targets, suggesting that RNA processing and phase separation can be partially uncoupled under certain conditions, although they may become more interdependent under stress or disease contexts^33,36^. Under pathological conditions, nuclear depletion and cytoplasmic aggregation of TDP-43, and associated splicing alterations are likely downstream consequences of earlier molecular events involving CR dysregulation or CR-driven LLPS, particularly given the high concentration of disease-associated mutations within and around the CR. Rather than targeting RNA splicing and other pathways disrupted by loss of nuclear TDP-43, targeting the CR may offer a more effective and potentially safe therapeutic strategy by addressing an upstream driver of TDP-43 toxicity.

Perhaps one of the most promising aspects of this study is the identification of a potent small molecule capable of binding TDP-43 to suppress its neurotoxicity without impacting nuclear localization and primary nuclear function. Although previous studies have identified several small molecules that suppress TDP-43 aggregation or condensation, few were designed to target TDP-43 directly. For example, riluzole, the approved drug for ALS, inhibits TDP-43 self-interaction^61^, yet it primarily targets the glutamatergic pathway^62,63^. This highlights a significant gap in current therapeutic developments for addressing the specific molecular mechanisms underlying TDP-43-associated neurodegeneration. XL20 demonstrates CR-dependent target engagement, as supported by biochemical and/or cellular target-engagement assays showing loss of interaction upon CR deletion or Trp334 mutation. Importantly, like CR deletion, XL20 does not appear to disrupt its core RNA regulatory function. This specificity is critical, as it reduces the risk of off-target effects that could lead to unwanted side effects. Single- and multiple-dose toxicology studies of XL20 have demonstrated no observable toxicity and good tolerance in vivo, which is promising for its potential as a therapeutic agent. The ability of XL20 to cross the blood–brain barrier is also noteworthy, as it ensures that the compound can reach the CNS. Our pharmacokinetic studies demonstrate that XL20 is rapidly absorbed and distributed, achieving micromolar concentrations in the brain and spinal cord at a 50 mg per kg body weight dose, consistent with the effective levels required for its protective effects observed in dose–response experiments. These results, combined with evidence of efficient CNS penetration and a favorable safety profile, establish XL20 as a promising candidate for the treatment of TDP-43-related pathologies.

XL20 not only significantly extends the lifespan and mitigates the behavioral deficits of p.Ala315Thr mice but also alleviates neuronal loss. The protective effect of XL20 extends beyond TDP-43 overexpression, showing efficacy in improving neuronal function in human motor neurons bearing mutant TDP-43. As the CR is a key regulator of TDP-43 LLPS and aggregation^19,33^, XL20’s ability to engage the CR enables it to abolish rare TDP-43 inclusions observed in p.Ala315Thr mice, similar to its effects on stress-induced TDP-43 aggregation in cells. Importantly, XL20 does not act as a general aggregation or phase-separation inhibitor, and CR deletion abolishes XL20-mediated neuroprotective and bioenergetic effects, collectively supporting a CR-specific mechanism of action rather than nonspecific cytoprotection. Disease-associated mutations may lead to aberrant LLPS, which in turn contributes to cytoplasmic accumulation of TDP-43 (ref. ^64^); indeed, XL20 also reduced TDP-43 mislocalization in both p.Ala315Thr and p.Gln331Lys models, further supporting the notion that TDP-43 aggregation and especially mislocalization may be downstream consequences of CR activity. It remains unclear why p.Ala315Thr mice or p.Gln331Lys motor neurons do not exhibit significant splicing changes. Nevertheless, while p.Ala315Thr and especially p.Gln331Lys models lack prominent TDP-43 aggregation and splicing defects, suggesting these are not required for neurotoxicity in these models^65^, XL20 engages the CR, a region critical for TDP-43 pathology formation, and effectively suppresses both self-aggregation in vitro and stress-induced aggregation in cells. Therefore, under disease-relevant conditions, targeting the CR by XL20 may modulate an upstream driver of TDP-43 pathology and provide a more targeted and effective treatment strategy than existing therapies like riluzole.

Genetic deletion or pharmacological engagement of the CR confers neuroprotection in the absence of widespread aggregation, nuclear clearance or RNA splicing deficits, indicating that these disease-relevant features may not be the dominant mechanism. This aligns with increasing evidence that cytoplasmic TDP-43 is a key driver of pathology, while nuclear loss of function may contribute in a context-dependent manner^12,13^. Instead, our findings suggest mitochondrial bioenergetics as a possible downstream mechanism of CR-targeted therapy. Notably, similarly to CR deletion, XL20 treatment enhanced mitochondrial respiratory function, particularly within complex I and its supercomplex, a pathway known to be impaired in ALS, Alzheimer’s disease and other neurodegenerative diseases^66^. TDP-43 has been shown to bind mitochondria-encoded *ND3* and *ND6* transcripts, disrupt their translation and impair complex I dysfunction^51^, whereas CR deletion upregulates *ND3*/*ND6* and restores mitochondrial function. Importantly, both CR deletion and XL20 treatment suppress the mitochondrial localization of TDP-43, supporting mitochondria as downstream effectors of CR modulation. The LLPS-deficient FYW-L mutant, located outside the CR, mimics the effects of CR deletion on mitochondria-associated TDP-43, suggesting that the CR may regulate mitochondrial function through LLPS-mediated mitochondrial localization of TDP-43. This notion is supported by the observed dynamic and specific association between TDP-43 condensates and mitochondria, which could be intensified under cellular or mitochondrial stresses. Similar interactions were also observed for other neurodegeneration-associated proteins. Therefore, while mitochondria may not be the driver of neurodegeneration, their consistent association with pathological protein condensates supports LLPS as a common mechanism mediating the mitochondrial localization of these pathological proteins and the resulting dysfunction^67–69^. Of note, mitochondrial TDP-43 has also been reported to activate cGAS–STING signaling^53^, suggesting that, beyond alleviating pathological protein aggregation, mislocalization and nuclear depletion-associated RNA splicing changes, additional mitochondrial downstream pathways may contribute to the neuroprotection effects of XL20 or CR deletion and should be considered in future development of XL20-related compounds.

In conclusion, this study demonstrates the feasibility of targeting the CR of TDP-43 as a therapeutic strategy for neurodegeneration. By targeting a domain essential for neurotoxicity without disrupting key nuclear functions, we show that TDP-43-associated neurodegeneration may be effectively suppressed. XL20 emerged as a lead compound with CR-dependent target engagement and robust functional efficacy. The combined biochemical, genetic and cellular evidence supports a consistent mechanism of action, and provides strong proof-of-concept for targeting the CR. Pharmacologically targeting such motifs may represent a promising and previously underexplored approach for treating TDP-43-associated neurodegenerative diseases.

## Methods

### Ethics

All animal experiments were performed in accordance with National Institutes of Health guidelines and approved by the Institutional Animal Care and Use Committee at the University of Arizona (protocol no. 2022-1010). Mice were housed under a 12-h light–12-h dark cycle at 22 ± 2 °C and 40–60% humidity with ad libitum access to food and water.

### Cell culture and generation of knockout cell lines

HEK293 cells (American Type Culture Collection) were cultured in DMEM (D7777, Sigma–Aldrich) containing 10% fetal bovine serum and 1% penicillin–streptomycin at 37 °C in 5% CO_2_. MFN2-, DRP1-, and MFF-knockout HEK293 cell lines were generated using the CRISPR–Cas9 PX459 V2.0 vector (Addgene). Single-guide RNA sequences targeting *MFN2*, *DRP1* and *MFF* were 5′-GCGAATTAAGCAGATTACGG-3′, 5′-GCTGCCTCAAATCGTCGTAG-3′ and 5′-TCACTCAGCGTAAGTACACG-3′, respectively. Monoclonal lines were isolated by limiting dilution and validated by immunoblotting. Transfections were performed using TransIT-293 reagent (MIR2704, Mirus Bio) in Opti-MEM (51200038, Thermo Fisher Scientific).

Primary cortical neurons were isolated from E18 C57BL/6J mouse embryos. Cortices were dissociated in HBSS (14170112), Hibernate E medium (A1247601) and 2% B27 (A3582801), digested with 0.25% trypsin (25200056), and resuspended in Opti-MEM containing DNase I (50 U ml^−1^; 18047019; all from Thermo Fisher Scientific). Neurons were plated on poly-D-lysine-coated and laminin-coated BioCoat coverslips or chamber slides (354086 or 354632, Becton Dickinson Biosciences) and maintained in Neurobasal medium (21103049) supplemented with 1% GlutaMAX (35050061, Thermo Fisher Scientific) and 2% B27. Neurons were transfected using Lipofectamine 2000 (11668500, Thermo Fisher Scientific). Neuronal survival was quantified at DIV6 to DIV12 in neurons expressing EGFP, EGFP–TDP-43 WT, ΔCR, ΔNLS or ΔNLS/ΔCR, with DIV12 counts normalized to DIV6.

Human iPS cell-derived motor neurons (10HU-014, iXCells Biotechnologies) were cultured in motor neuron medium (MD-0022-100ML, iXCells Biotechnologies) on laminin/poly-L-ornithine-coated 96-well glass plates. At DIV14, neurons were treated with vehicle (DMSO) or XL20 (20 µM) for 7 days.

### Expression vectors and chemicals

pDsRed2-ER and pDsRed2-Mito were purchased from Clontech. TagRFP-Endo and TagRFP-Golgi were obtained from Evrogen. TagRFP-Lyso was generated by cloning TagRFP and TMEM119 into pcDNA3.1(+) vectors (Invitrogen). EGFP-tagged or 4×Flag-tagged TDP-43 WT and mutant constructs, as well as EGFP-tagged UBQLN2, SQSTM1 and FUS constructs, were generated by Gibson assembly using pcDNA3.1(+) vectors. For mitochondrial targeting, the mAKAP1 targeting sequence (amino acids 34–63) was fused to FKBP12 and tagged with TagGFP or Myc. For endoplasmic reticulum targeting, TagRFP-tagged or Flag-tagged FRB constructs were fused to the targeting sequence of Sac1 protein.

NaAsO_2_ (S7400), sorbitol (56755) and antimycin A (A8674) were purchased from Sigma–Aldrich. CCCP (0452/500) was obtained from Tocris. TMRM (T668) was purchased from Thermo Fisher Scientific. AP21967 (635057) was obtained from Takara Bio. XL20 (BDF34019555) was identified from the Asinex compound library and synthesized by WuXi AppTec. Newly generated plasmids and related reagents are available from the corresponding author upon reasonable request.

### RNA extraction and RT–qPCR

Total RNA was extracted using the AllPrep DNA/RNA/miRNA Universal Kit (80224, Qiagen). cDNA was synthesized using High-Capacity cDNA Reverse Transcription Kits (4368814, Applied Biosystems). RT–qPCR was performed using SYBR Premix Ex Taq (RR420A, Takara Bio) on a CFX96 real-time PCR system (Bio-Rad Laboratories) with cycling conditions of 95 °C for 30 s followed by 40 cycles of 95 °C for 5 s and 60 °C for 30 s. Relative mRNA levels were normalized to GAPDH using comparative Ct analysis. Primer sequences are listed in Supplementary Table 1.

### Thioflavin T fluorescence assay

Recombinant proteins were expressed in BL21-Gold (DE3) cells and purified using the Ni-NTA Fast Start Kit (30600, Qiagen). Thioflavin T (ThT) assays were performed using 10 µM recombinant proteins in 20 mM MES buffer (pH 5.5) containing 20 µM ThT with DMSO or XL20. Tau aggregation assays used monomeric Tau protein (0.5 mg ml^−1^; SPR-329, StressMarq) with heparin (50 µg ml^−1^), whereas Aβ_1–42_ fibrillization assays used 10 µM HFIP-dissolved Aβ_1–42_ in PBS containing 20 µM ThT with DMSO or XL20. Samples were incubated in black 96-well plates (Corning) at 37 °C with shaking, and fluorescence was measured every 5 min for at least 12 h using a Synergy H1 microplate reader (BioTek) at excitation and emission wavelengths of 446 nm and 482 nm, respectively.

### Immunoblotting, immunocytochemistry, immunofluorescence and Nissl staining

Mouse tissues or HEK293 cells were lysed in buffer containing 20 mM Tris-HCl (pH 7.5), 150 mM NaCl, 1% Triton X-100, PMSF (10837091001, Sigma–Aldrich), leupeptin (9803, Cell Signaling Technology) and protease/phosphatase inhibitor cocktails (P8340 and P0001, Sigma–Aldrich). Proteins (20 µg) were separated by 10% SDS–PAGE, transferred to polyvinylidene fluoride membranes and developed using Immobilon Western Chemiluminescent HRP Substrate (WBKLS0050, Millipore). Images were acquired using an iBright CL1500 Imaging System (Thermo Fisher Scientific).

Immunocytochemistry was performed on 6-µm paraffin sections following antigen retrieval with Antigen Decloaker (Biocare), blocking in 10% normal goat serum and overnight incubation with primary antibodies at 4 °C. Signals were detected using species-specific secondary antibodies and peroxidase–anti-peroxidase complexes (Jackson ImmunoResearch Laboratories) with 3,3′-diaminobenzidine.

For immunofluorescence, tissue sections or fixed cells were incubated with Alexa Fluor-conjugated secondary antibodies (Thermo Fisher Scientific), counterstained with DAPI and mounted using Fluoromount-G (0100-01, SouthernBiotech). Nissl staining was performed on paraffin-embedded brain and lumbar spinal cord sections using 0.1% Cresyl Violet. Primary antibodies are listed in Supplementary Table 2.

### Mitochondrial fractionation and high-resolution respirometry

Live cells or frozen tissues were homogenized in IB-1 buffer (225 mM mannitol, 75 mM sucrose, 0.1 mM EGTA and 20 mM HEPES, pH 7.4). Following sequential centrifugation at 600*g* and 7,000*g*, mitochondrial pellets were washed in IB-2 buffer (225 mM mannitol, 75 mM sucrose and 20 mM HEPES, pH 7.4) and resuspended in mitochondrial respiration buffer (250 mM mannitol, 0.5 mM EGTA and 5 mM HEPES, pH 7.4). Highly enriched mitochondria were purified using 30% Percoll gradients and ultracentrifugation at 95,000*g* for 30 min at 4 °C using an Optima L-100 XP ultracentrifuge (Beckman Coulter). Mitochondrial OCR was measured using an Oxygraph-2k respirometer (Oroboros Instruments) with isolated mitochondria (50 µg) or intact cells (5 × 10^5^ cells). OCR values were normalized to mitochondrial protein or cell number. Malate, pyruvate, glutamate, succinate, adenosine diphosphate, cytochrome c, carbonyl cyanide-p-trifluoromethoxyphenylhydrazone, rotenone, antimycin A, *N*,*N*,*N*′,*N*′-tetramethyl-p-phenylenediamine dihydrochloride, ascorbate, palmitoylcarnitine, digitonin, tetramethylhydroquinone and azide were sequentially added. OCR recordings were analyzed using DatLab software (Oroboros Instruments).

### Lumbar spinal cord injection of AAV

WT C57BL/6J mice (strain no. 000664, Jackson Laboratory) received lumbar spinal cord injections of AAV1 vectors encoding EGFP, TDP-43 WT, ΔNLS or ΔNLS/ΔCR under the SYN promoter (VectorBuilder). Three-month-old mice were anesthetized with isoflurane and positioned in a stereotactic frame on a heating pad. Following local bupivacaine administration (5 mg ml^−1^), lumbar vertebrae L1–L3 were exposed by laminectomy. AAV1 (1.5 µl per site) was bilaterally injected into the lumbar spinal cord using a Hamilton syringe at a depth of 1 mm and 1 mm lateral to the midline. Injections were delivered over 2 min followed by a 5-min diffusion period. Fascia and skin were sutured, and mice received carprofen (100 mg ml^−1^) for two consecutive days with postoperative monitoring, including paralysis assessment.

### Small-molecule administration

Prnp-TARDBP^p.Ala315Thr^ mice (p.Ala315Thr mice; 010700, Jackson Laboratory) and non-carrier littermates on a C57BL/6 background were used for small-molecule treatment studies. XL20 was initially dissolved in DMSO and further diluted in corn oil containing 10% DMSO to a final concentration of 4 mg ml^−1^ before administration. Mice received i.p. injections of XL20 at 50 mg per kg body weight every 3 days. Control mice received vehicle injections containing the same corn oil/10% DMSO solution at the same dosing schedule and volume.

### Behavioral tests

Behavioral analyses included rotarod, grip strength, tail suspension and footprint assays performed with investigators blinded to treatment. Mice underwent rotarod and grip strength testing on days 1–3 and tail suspension testing on days 4–6. For rotarod analyses, mice received two training trials per day for 3 days, followed by testing on an accelerating rotarod (4–40 rpm over 5 min) and maximal latency to fall was recorded. Grip strength was measured using a grip strength meter (Bioseb), and the best value from five trials was used for analysis. For tail suspension tests, mice were suspended for 6 min, and latency to immobility and total immobility time were scored by two blinded observers. Mice were considered immobile when motionless for at least 2 s. Footprint analyses were performed using a customized runway (50 cm × 5 cm), and stride length was quantified as the mean forward distance between consecutive steps. Behavioral analyses were performed using ANY-maze software (Stoelting).

### Electromyography recordings of skeletal muscles

Compound muscle action potentials evoked by sciatic nerve stimulation were recorded using a PowerLab 4/35 system with FE155 Stimulator HC and FE136 Animal Bio Amp (ADInstruments) and ring electrodes (Natus Neurology). Mice were anesthetized with isoflurane, and electrodes were positioned at the gastrocnemius muscle and sciatic nerve. Electrical stimuli (1–10 mV, 0.1 ms duration) were delivered at 10 Hz.

### Virtual screening and molecular docking

Virtual screening was performed using the Maestro software suite (Schrödinger). TDP-43 receptor models were prepared from AlphaFold-predicted structures and the published TDP-43 structure (Protein Data Bank, PDB 8GCC). The Asinex Biodesign library containing 190,337 compounds was prepared using LigPrep, which generates low-energy 3D ligand structures suitable for Glide docking. TDP-43 structures were prepared using the Protein Preparation Wizard, including removal of water molecules, termini capping, assignment of bond orders and formal charges, and optimization of hydroxyl, Asn, Gln and His states using ProtAssign/Epik. These preparation steps are standard components of the Schrödinger docking workflow. Docking was performed using Glide with the OPLS4e force field. Compounds were filtered using Lipinski’s Rule and QikProp criteria, excluding molecules with molecular weight >500 Da, >10 hydrogen bond acceptors, >5 hydrogen bond donors or log*P* > 5. Residues 320–340 of TDP-43 were selected as the target region, and receptor grid boxes were generated around these residues. Docking was performed sequentially using HTVS, SP and XP modes with Epik state penalties included during scoring. Compounds with docking scores < −6.0 kcal mol^−1^ were subjected to Induced-Fit Docking using the recommended Maestro workflow to account for local receptor flexibility and refine ligand–protein interactions. Final docking scores, binding poses and interaction interfaces were analyzed using the Maestro Ligand Interaction tool to prioritize candidate TDP-43 CR modulators for experimental validation.

### SPR

SPR experiments were performed using a Biacore 1 K system (GE Healthcare Life Sciences). Recombinant full-length TDP-43 proteins were purified under denaturing conditions (GenScript) and immobilized onto CM5 sensor chips (Cytiva) by amine coupling in 10 mM acetate buffer (pH 4.0), yielding approximately 4,000 RUs. CR peptides synthesized by Biotak were immobilized at approximately 5,000 RUs. Binding assays were performed in PBS-P buffer (0.2 M phosphate buffer, 137 mM NaCl, 2.7 mM KCl, 0.05% P20 and 5% DMSO, pH 7.4). XL20 was injected at 3.125–50 µM with 120-s association and 180-s dissociation phases. Sensor surfaces were regenerated using 1 M NaCl. Binding responses were analyzed using a 1:1 interaction model in BIAevaluation software (GE Healthcare Life Sciences). Because sensorgrams did not reach steady-state equilibrium and deviated from ideal single-site kinetics, affinity values were interpreted as approximate estimates for qualitative comparison.

### Cell lysate thermal shift assay

Forty-eight hours after transfection, cell pellets (2 × 10^7^ cells) were resuspended in 1 × PBS containing protease and phosphatase inhibitor cocktails and lysed by three freeze–thaw cycles in liquid nitrogen. Clarified lysates were collected by centrifugation at 18,000*g* for 20 min at 4 °C and aliquoted into PCR tubes. Lysates were incubated with XL20 (20 µM final concentration) or DMSO for 30 min at room temperature and heated to 44–68 °C in 3 °C increments for 3 min using a thermocycler, followed by cooling to 20 °C for 1 min. Samples were then centrifuged at 20,000*g* for 20 min at 4 C, mixed with Laemmli buffer, heated at 95 °C for 5 min and analyzed by immunoblotting.

### Pharmacokinetic analysis

XL20 was administered i.p. at doses of 5 or 50 mg per kg body weight (*n* = 3 mice per group). Blood, CSF, brain and spinal cord samples were collected at 0.083 h, 0.25 h, 0.5 h, 1 h, 2 h, 4 h, 8 h and 24 h after administration. Following protein precipitation, XL20 concentrations were quantified by liquid chromatography–tandem mass spectrometry (LC–MS/MS) using a Triple Quad 5500 system (SCIEX), and data were analyzed using Analyst Software v1.6.3 (SCIEX).

### Microscopy, live imaging and FRAP

Confocal imaging was performed using a Leica STELLARIS 5 microscope (Leica) equipped with a HC Plan-Apo ×63/1.4-NA oil immersion objective, STELLARIS 5 W diode lasers, 405 DMOD lasers and Power HyD detectors. Live-cell imaging was conducted using a stage-top incubator (Tokai Hit) maintained at 37 °C with controlled CO_2_ and humidity. For mitochondria and protein condensate imaging, cells or neurons cultured on 35-mm glass-bottom dishes were labeled with MitoTracker Red CMXRos for 30 min. Time-lapse images were acquired every 1.76 s for 5 min. Initial image processing was performed using LAS X software (Leica), and condensate–mitochondria interactions were analyzed using ImageJ.

For mitochondrial membrane potential imaging, primary cortical neurons were transfected at DIV5. At DIV8, neurons were incubated with TMRM for 30 min followed by a 10-min equilibration period before live imaging. Only EGFP-positive neurons were included for analysis. FRAP experiments were performed using a ×63/1.4-NA oil objective. Following acquisition of five baseline images, bleaching was performed using a 488-nm laser line at 100% intensity for ten iterations. Fluorescence recovery was monitored every 1.76 s for 2 min. FRAP data were analyzed using LAS X software.

### Image analysis

Three-dimensional image reconstruction and analyses were performed using Imaris 7 (Oxford Instruments), Fiji/ImageJ, Image-Pro Plus 6.0 (Media Cybernetics) and GraphPad Prism 8 (GraphPad Software). Confocal *z*-stack images were subjected to uniform background subtraction, contrast adjustment and intensity thresholding before generation of 3D renderings and binary masks for TDP-43 condensates and mitochondria. Identical threshold, size and circularity parameters were applied across all experimental groups. Condensates were classified as mitochondria-associated when voxel overlap with mitochondrial masks was detected in three-dimensional space. The percentage of mitochondria-associated condensates was calculated by normalizing overlapping condensates to the total number of condensates per cell. Cells lacking detectable condensates after thresholding were assigned a value of zero. TDP-43ΔNLS/ΔCR-expressing and ΔNLS/FYW-L-expressing cells exhibited predominantly diffuse cytosolic localization with few detectable condensates and were therefore largely scored as zero. For kymograph analyses, live-cell imaging sequences were processed in Fiji/ImageJ using the reslice function to quantify interaction duration and movement distances between mitochondria and TDP-43 condensates. Mitochondrial length was quantified as maximal Feret diameter following threshold-based segmentation, and NeuN-positive neuronal counts were quantified automatically using Image-Pro Plus. Quantitative comparisons were performed using samples processed and imaged in parallel under identical acquisition and analysis settings.

### RNA-sequencing and transcriptomic analysis

Total RNA extracted using Qiagen RNA extraction kits (Qiagen) was submitted to Inomics for library preparation and sequencing. RNA quality was assessed using an Agilent 4150 Bioanalyzer (Agilent Technologies). Raw sequencing reads underwent quality control and filtering before alignment to the mouse reference genome using HISAT2. Clean reads were mapped to the reference transcriptome using Bowtie2 (v2.3.4.3), and transcript abundance was quantified using RSEM (v1.3.1). Differential gene expression analyses were conducted using DESeq2 (v1.4.5), DEGseq or PoissonDis with significance thresholds of *Q* ≤ 0.05 or false discovery rate (FDR) ≤ 0.001. Differential splicing analyses were performed using thresholds of *P* ≤ 0.05 or FDR ≤ 0.01.

### Synaptosome enrichment and mass spectrometry analysis

Mouse brains were homogenized in ice-cold buffer (0.32 M sucrose, 4 mM HEPES, pH 7.4, 1 µM PMSF and protease/phosphatase inhibitors) and sequentially centrifuged at 1,000*g* and 12,500*g* to obtain crude synaptosomal pellets. P2 fractions were purified using a 0.8 M/1.2 M sucrose gradient followed by ultracentrifugation at 50,000*g* for 60 min at 4 °C using an SW55 Ti rotor (Beckman Coulter). Synaptosome-enriched fractions were lysed in NP-40-containing buffer with protease/phosphatase inhibitors. Proteins (20 µg) were separated on 4–20% Mini-PROTEAN TGX gels (Bio-Rad Laboratories), subjected to in-gel Lys-C digestion, and analyzed by LC–MS/MS using a timsTOF Pro2 Q-TOF system with a CaptiveSpray ion source (Bruker Daltonics). Peptides were separated on a 15-cm × 75-µm C18 column using an acetonitrile/0.1% formic acid gradient and analyzed by parallel accumulation–serial fragmentation acquisition. Proteins were identified against the UniProt mouse database using Andromeda in MaxQuant v2.7.3.0 with carbamidomethylation as a fixed modification and methionine oxidation and N-terminal acetylation as variable modifications. Searches allowed up to two missed cleavages with an FDR cutoff of 0.01. Label-free quantification and downstream analyses were performed using Perseus software with log_2_-transformed label-free quantification values and normal distribution-based missing-value imputation.

### Statistics and reproducibility

Sample sizes were determined by power analysis to detect a 20% difference with *α* = 0.05 and power = 0.8. No samples were excluded from the analyses. No method of randomization was used to assign animals or samples to experimental groups. Behavioral assessments were performed in a blinded manner; investigators conducting the behavioral tests were unaware of both treatment allocation and genotype. Other experiments and analyses were not performed under blinded conditions unless otherwise stated. Statistical analyses were conducted using GraphPad Prism 8 with one-way or two-way ANOVA followed by Tukey’s multiple-comparison test, Dunnett’s multiple-comparisons test or two-sided unpaired Student’s *t*-test, as indicated in the figure legends. Exact *n* values, statistical tests and multiple-comparison adjustments are provided in the figure legends. Data are presented as the mean ± s.e.m. unless otherwise stated. Technical replicates were not treated as independent biological replicates. Representative experiments were independently repeated at least three times with similar results unless otherwise indicated. Data distribution and variance assumptions were assessed before statistical testing. *P* < 0.05 was considered statistically significant.

### Reporting summary

Further information on research design is available in the Nature Portfolio Reporting Summary linked to this article.

## Supplementary information

Supplementary Information Supplementary Figs. 1–5 and Supplementary Tables 1 and 2.

Reporting Summary

Supplementary Video 1 Tail suspension tests of 5-month-old male WT mice injected with AAV1-Syn-EGFP, ∆NLS or ∆NLS/CR at 3 months old.

Supplementary Video 2 Moving tests of 5-month-old male WT mice injected with AAV1-Syn-EGFP, ∆NLS or ∆NLS/CR at 3 months old.

Supplementary Video 3 Tail suspension tests of 70-day-old NTG and A315T male mice, which had been treated with either a vehicle or XL20 starting from the age of 45 days.

Supplementary Video 4 Time-lapse imaging of TDP-43 condensates (green) and endoplasmic reticulum (labeled by DsRed-ER, purple) in cells transfected with EGFP–TDP-43∆NLS. Six frames per second.

Supplementary Video 5 Time-lapse imaging of TDP-43 condensates (green) and lysosome (labeled by TagRFP-lysosome, purple) in cells transfected with EGFP–TDP-43∆NLS. Six frames per second.

Supplementary Video 6 Time-lapse imaging of TDP-43 condensates (green) and mitochondria (labeled by MitoTracker, purple) in cells transfected with EGFP–TDP-43∆NLS. The rectangle marks the ‘kiss-and-run’ events between TDP-43 condensates and mitochondria. Six frames per second.

## Source data

Source Data Figs. 1–6 and Extended Data Figs. 1–10 Statistical source data.

Source Data Figs. 1, 2, 4 and 6 and Extended Data Figs. 1, 3–6, 9 and 10 Unprocessed western blots and/or gels.

## Data Availability

The mass spectrometry proteomics data generated in this study have been deposited in the ProteomeXchange Consortium via the PRIDE partner repository under accession code PXD060626. Bulk RNA-sequencing data have been deposited in the Sequence Read Archive under accession code PRJNA1220042. All other data supporting the findings of this study are available within the paper and its Supplementary Information or from the corresponding author upon reasonable request. Source data are provided with this paper.
