# Supplementary material for: Therapeutic targeting of the conserved region within the low-complexity domain of TDP-43 is neuroprotective and extends survival in amyotrophic lateral sclerosis mice
Source: Nat Aging. 2026 Jul 3;6(8):1667–83. doi: 10.1038/s43587-026-01166-3 (PMC13472882; doi:10.1038/s43587-026-01166-3)
Supplement: Supplementary file 1 [file 43587_2026_1166_MOESM10_ESM.pdf]

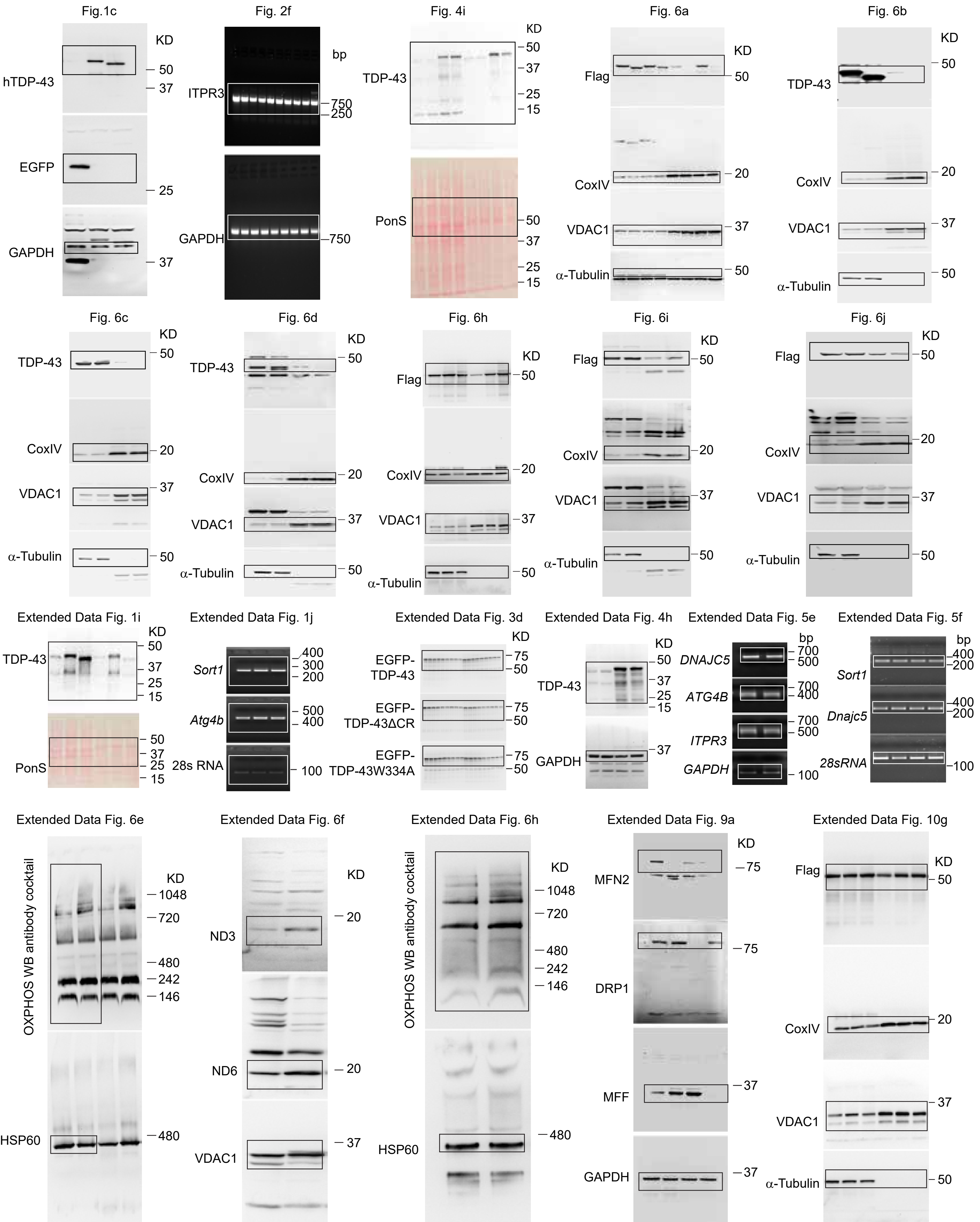

Fig.1c
KD
hTDP-43
50
37
EGFP
25
GAPDH
37
Fig. 2f
bp
ITPR3
750
250
GAPDH
750
Fig. 4i
KD
TDP-43
50
37
25
15
PonS
50
37
25
15
Fig. 6a
KD
Flag
50
CoxIV
20
VDAC1
37
α-Tubulin
50
Fig. 6b
KD
TDP-43
50
CoxIV
20
VDAC1
37
α-Tubulin
50
Fig. 6c
KD
TDP-43
50
CoxIV
20
VDAC1
37
α-Tubulin
50
Fig. 6d
KD
TDP-43
50
CoxIV
20
VDAC1
37
α-Tubulin
50
Fig. 6h
KD
Flag
50
CoxIV
20
VDAC1
37
α-Tubulin
50
Fig. 6i
KD
Flag
50
CoxIV
20
VDAC1
37
α-Tubulin
50
Fig. 6j
KD
Flag
50
CoxIV
20
VDAC1
37
α-Tubulin
50
Extended Data Fig. 1i
KD
TDP-43
50
37
25
15
PonS
50
37
25
15
Extended Data Fig. 1j
Sort1
400
300
200
Atg4b
500
400
28s RNA
100
Extended Data Fig. 3d
KD
EGFP-TDP-43
75
50
EGFP-TDP-43ΔCR
75
50
EGFP-TDP-43W334A
75
50
Extended Data Fig. 4h
KD
TDP-43
50
37
25
15
GAPDH
37
Extended Data Fig. 5e
bp
DNAJC5
700
500
ATG4B
700
400
ITPR3
700
500
GAPDH
100
Extended Data Fig. 5f
bp
Sort1
400
200
Dnajc5
400
200
28sRNA
100
Extended Data Fig. 6e
OXPHOS WB antibody cocktail
KD
1048
720
480
242
146
HSP60
480
Extended Data Fig. 6f
KD
ND3
20
ND6
20
VDAC1
37
Extended Data Fig. 6h
OXPHOS WB antibody cocktail
KD
1048
720
480
242
146
HSP60
480
Extended Data Fig. 9a
KD
MFN2
75
DRP1
75
MFF
37
GAPDH
37
Extended Data Fig. 10g
KD
Flag
50
CoxIV
20
VDAC1
37
α-Tubulin
50
