# Supplementary material for: Therapeutic targeting of the conserved region within the low-complexity domain of TDP-43 is neuroprotective and extends survival in amyotrophic lateral sclerosis mice
Source: Nat Aging. 2026 Jul 3;6(8):1667–83. doi: 10.1038/s43587-026-01166-3 (PMC13472882; doi:10.1038/s43587-026-01166-3)
Supplement: Supplementary file 2 [file 43587_2026_1166_MOESM1_ESM.pdf]

nature aging

Supplementary information

https://doi.org/10.1038/s43587-026-01166-3

Therapeutic targeting of the conserved region within the low-complexity domain of TDP-43 is neuroprotective and extends survival in amyotrophic lateral sclerosis mice

In the format provided by the authors and unedited

a

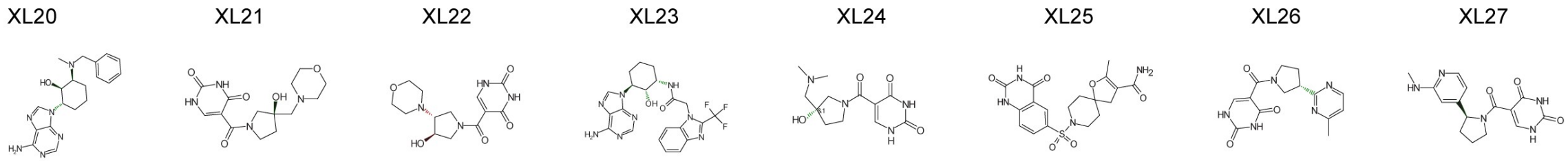

b

Wanglab ID	Asinex ID	Docking Score (kcal/mol)	XP Gscore (kcal/mol)	Glide Emodel (kcal/mol)
XL24	BDE31265220	-8.513	-8.698	-44.930
XL22	BDE32165749	-8.493	-9.107	-49.921
XL21	BDE32163394	-8.444	-9.155	-54.515
XL20	BDF34019555	-8.319	-8.349	-47.798
XL23	LAS51502065	-8.277	-8.277	-50.388
XL26	BDE32053214	-8.154	-8.801	-55.519
XL25	BDF33572449	-8.041	-8.134	-62.530
XL27	BDE32081987	-7.986	-8.168	-57.562

c

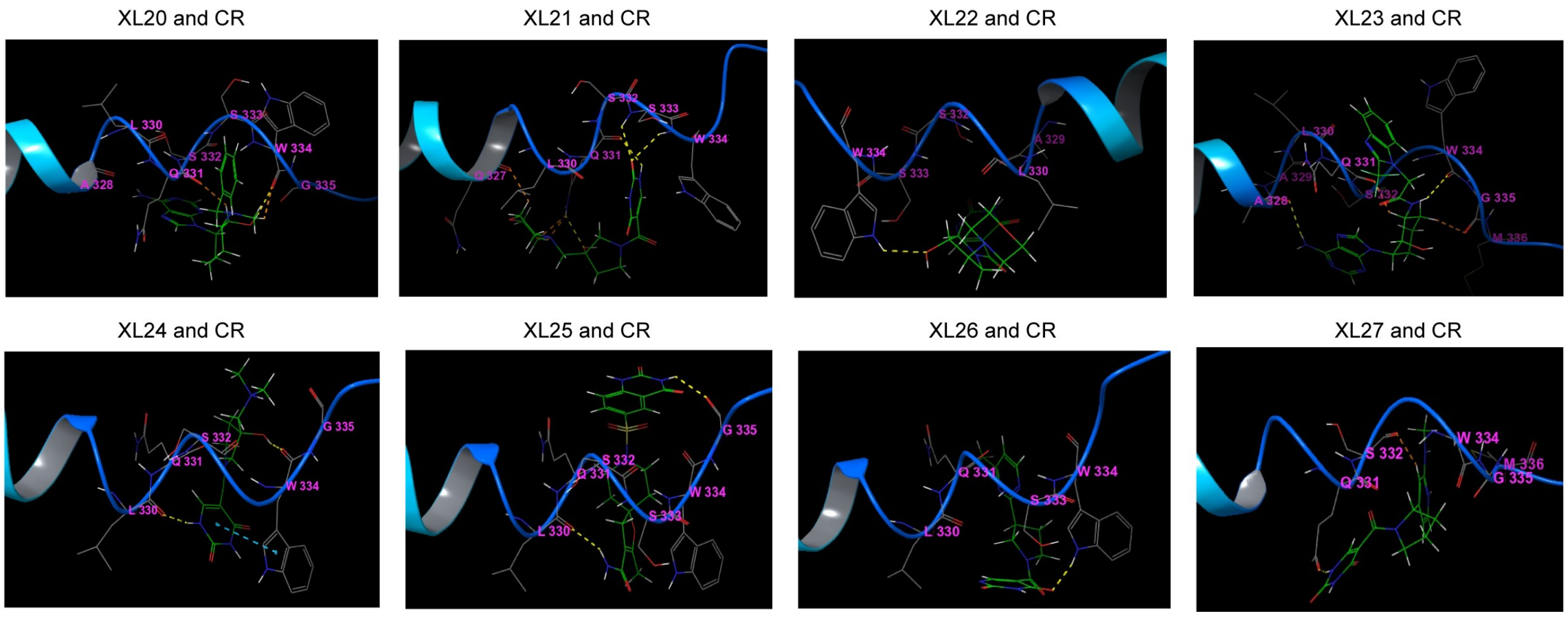

Supplementary Figure 1. Identification and validation of small molecules binding CR. (**a**) Molecular structures of the top small molecules interacting with the TDP-43 CR domain. (**b**) The list of top 8 hits with the lowest docking score. (**c**) 3D view of protein–ligand interactions of TDP-43 CR and XL20-27. The major amino acid residues involved in interactions between the CR and all 8 hit small molecules are shown. XL20 binds to the CR by engaging residues A328–G335 within a flexible loop adjacent to a short helical segment. Binding is anchored by Trp334 through hydrophobic and aromatic interactions, with additional stabilization provided by polar contacts involving Q331, S332, and S333. Importantly, the contact maps reflect dynamic rearrangements within the CR region rather than an unfolding artifact, indicating a flexible, conformation-dependent binding mode.

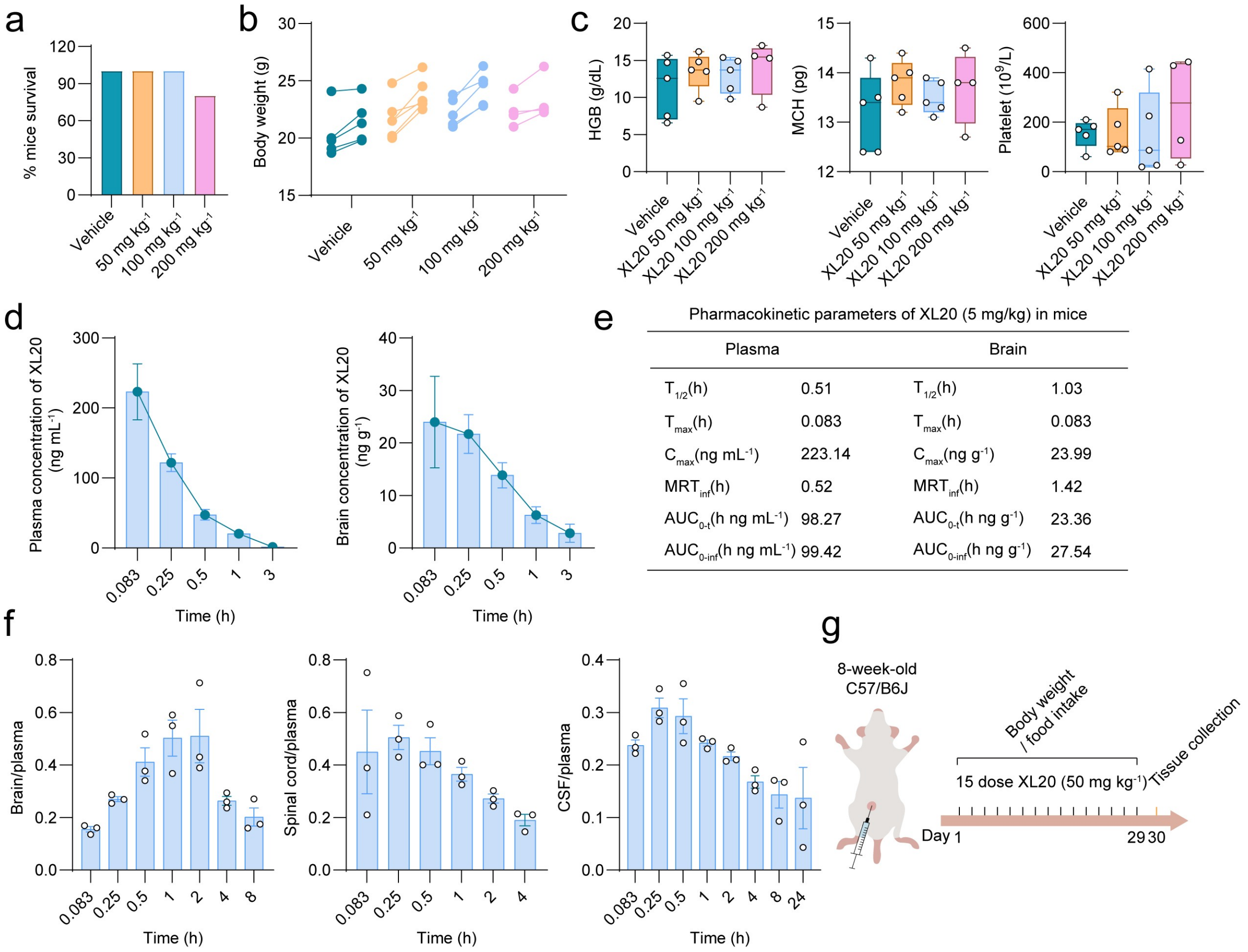

Supplementary Figure 2. *In vivo* toxicity of XL20. (**a**–**c**) Percentage of survival (**a**), body weight (**b**), and complete blood count (**c**) of female WT mice injected with vehicle and XL20 at different dose (*n* = 4–6 mice per group). (**d**,**e**) Pharmacokinetic parameters of XL20 following IP injection at a dose of 5 mg kg^{-1}. (**f**) Brain, spinal cord and CSF concentration of XL20 after normalized to plasma concentration (*n* = 3 per group). (**g**) Schematic diagram of the drug administration strategy for 8-week-old C57/B6J mice treated with repeated doses of XL20 at 50 mg kg^{-1} (*n* = 7–9 mice per group). Data are means ± SEM of triplicate independent experiments. Box-and-whisker plots show the median, interquartile range and min–max values; dots indicate individual data points. One-way ANOVA followed by Tukey's multiple comparison test.

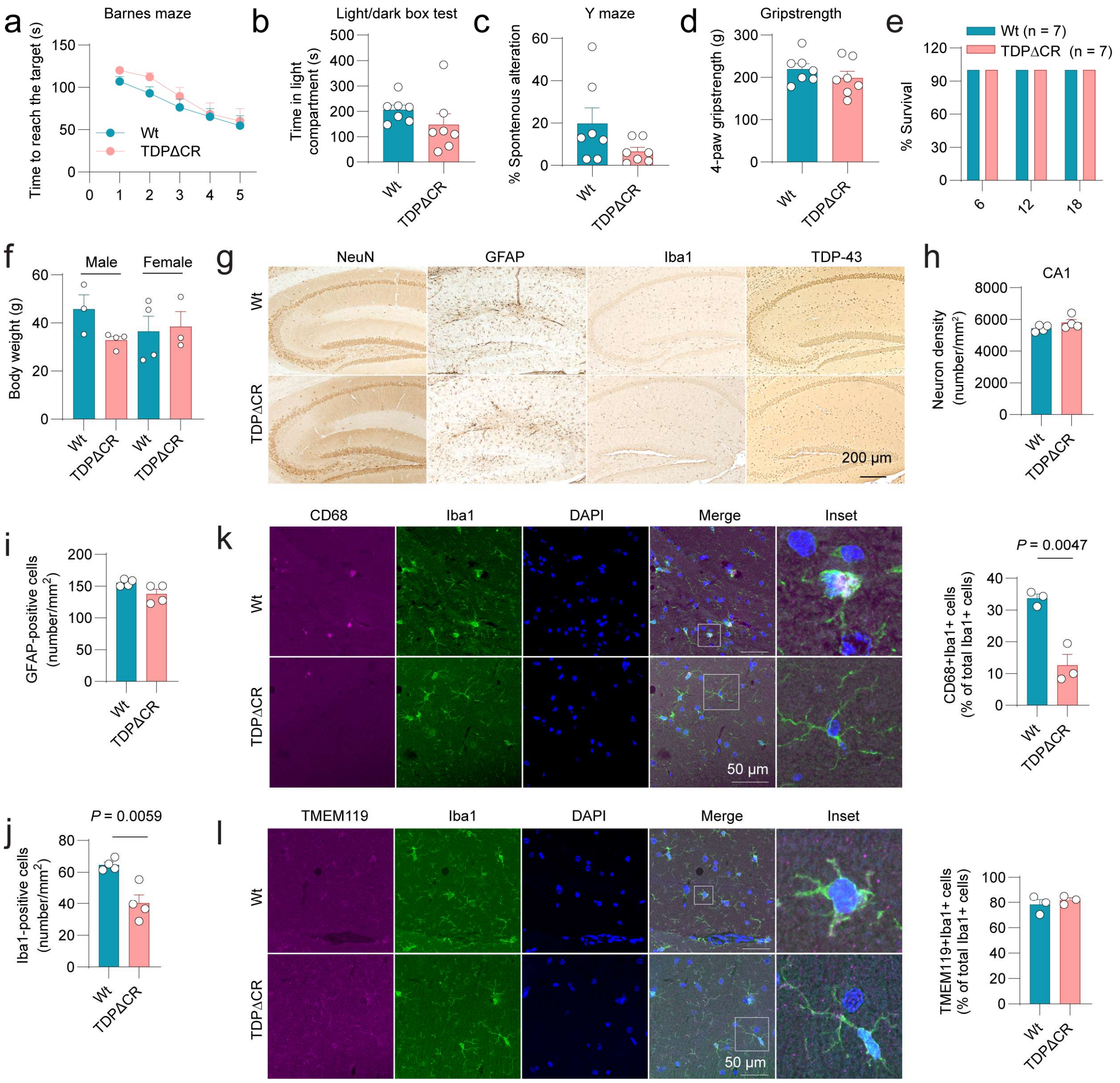

Supplementary Figure 3. Characterization of TDPΔCR mice at 18 months of age. (**a–d**) The tests included cognitive assessments (Barnes maze, **a,** *n* = 9–10 mice; Y-maze, **c**, *n* = 7 mice), anxiety tests (light/dark box, **b**; *n* = 7 mice), and motor function evaluations (grip strength test, **d**; *n* = mice). At this advanced age, no cognitive or motor deficits were observed in TDPΔCR mice compared to wild-type (WT) mice. (**e–f**) Percentage of survival (**e**), body weight (**f**), and representative immunohistochemistry staining of TDP-43 (**g**) in 18-month-old Wt and TDPΔCR mice. (**h–j**) Cell count for NeuN- (**h**), GFAP- (**i**), and Iba1-positive (**j**) cells in g (*n* = mice per group). (**k** and **l**) Representative images and quantification of CD68- (**k**) and TMEM119-positive (**l**) microglial cells in 18-month-old WT and TDPΔCR mice (*n* = 3 mice per group). Data are means ± SEM from independent experiments. One- or Two-way ANOVA followed by Tukey's multiple comparison test, or two-sided unpaired Student's *t*-test.

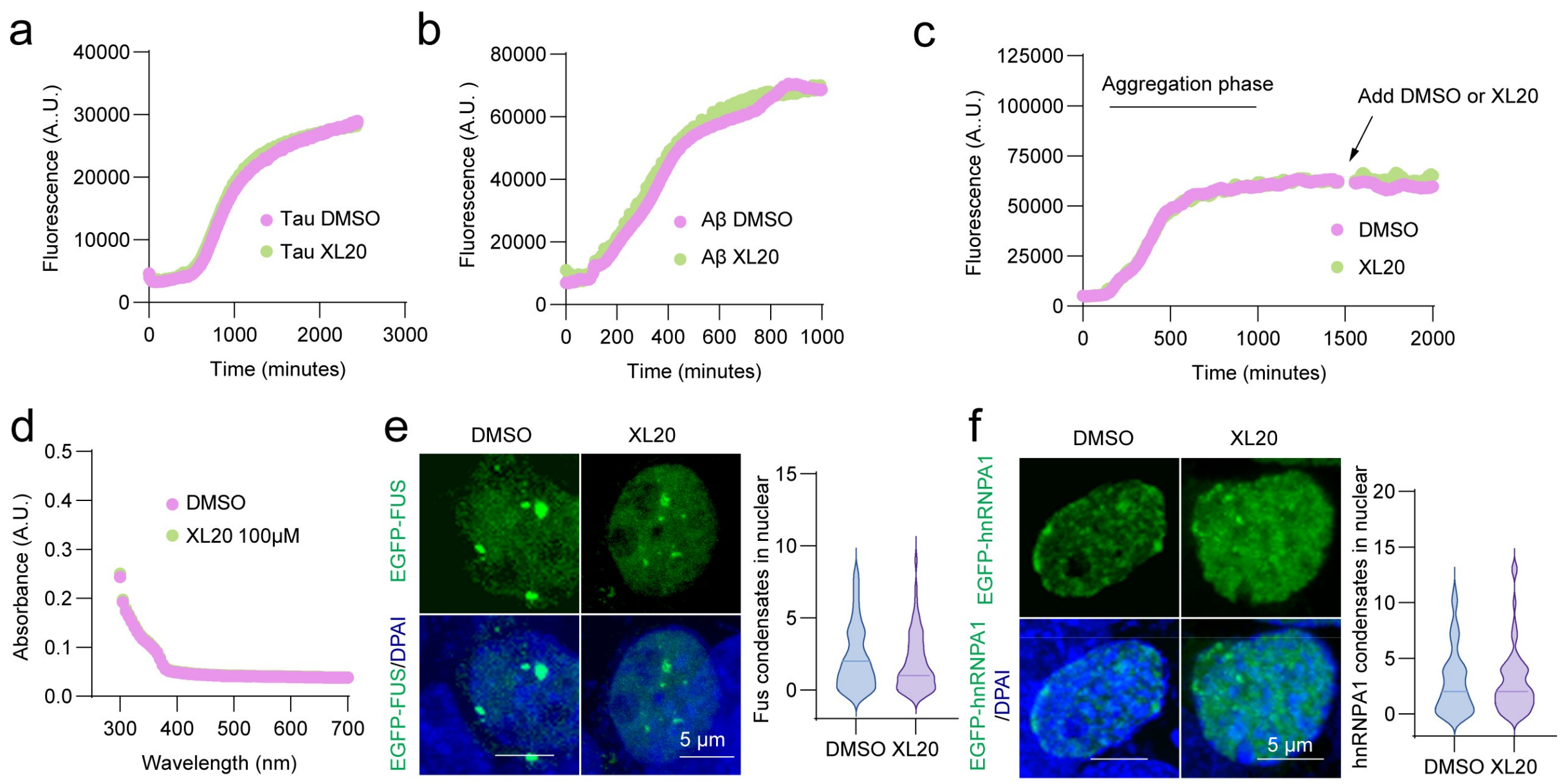

Supplementary Figure 4. XL20 has no effect on the aggregation or LLPS of other neurodegeneration-related proteins. (**a** and **b**) ThT assays of full-length Tau (**a**) and Aβ1-42 (**b**) incubated with DMSO or XL20 (100 μM) (*n* = 3–4 replicates). (**c**) XL20 does not affect ThT fluorescence in preformed Aβ aggregates. Aβ1–42 aggregation was initiated and allowed to reach the plateau phase before addition of DMSO or XL20 (100 μM). XL20 did not alter ThT fluorescence signals compared to DMSO, indicating no detectable effect on fluorescence readout under these conditions. (**d**) UV–Vis absorption spectrum of XL20 (100 μM). XL20 exhibits strong absorbance below 300 nm and minimal absorbance above 300 nm, with the spectrum closely overlapping that of the DMSO control. These results indicate no appreciable interference with fluorescence measurements in the visible range, including ThT excitation (446 nm) and emission (482 nm) used in this study. (**e** and **f**) Representative immunofluorescence images and quantification of FUS (**e**) and hnRNPA1 (**f**) condensates in HEK293 cells transfected with EGFP–FUS or EGFP–hnRNPA1 and treated with DMSO or XL20 (100 μM) (*n* = 53–58 cells). Data are means ± SEM. Two-sided unpaired Student's *t*-test.

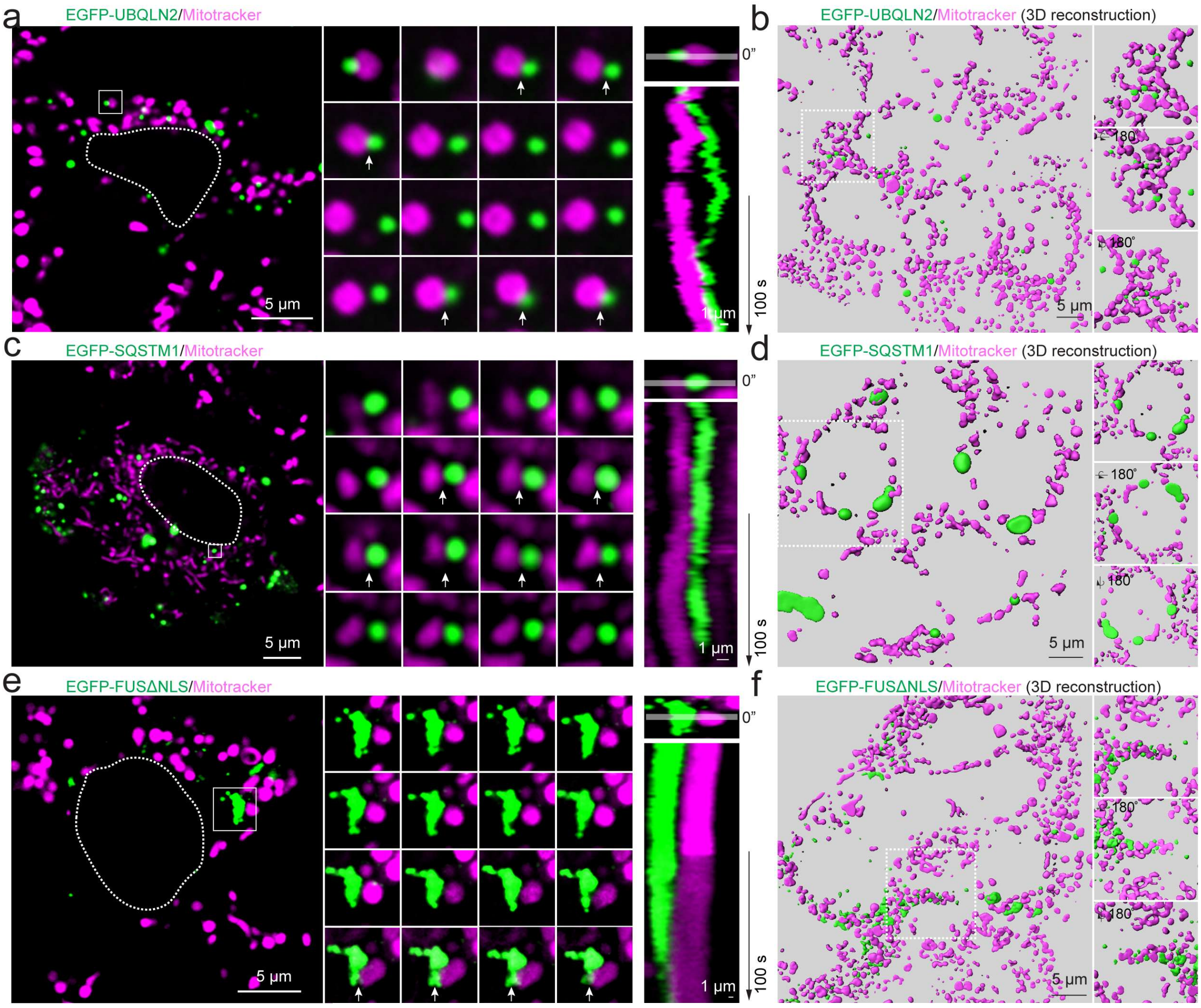

Supplementary Figure 5. Dynamic interactions between other neurodegeneration-related protein condensates and mitochondria. (**a**, **c**, and **e**) Representative large-field (left panel), enlarged time-lapse images (middle panel), and kymographs (right panel) of TDP-43 condensates (green) and mitochondria (purple) in cells transfected with EGFP-UBQLN2 (**a**), EGFP-SQSTM1 (**c**), or EGFP-FUSΔNLS (**e**). Dashed circles indicate the nucleus, and the arrowheads mark the “kissing” events. (**b**, **d**, and **f**) Representative 3D images of TDP-43 condensates (green) associated with mitochondria (purple) in cells transfected with EGFP-UBQLN2 (**b**), EGFP-SQSTM1 (**d**), or EGFP-FUSΔNLS (**f**). Adjacent to the 3D image, cropped images show the selected region rotated 180 degrees either horizontally or vertically.

Primer name	DNA sequence (5'-3')
hATG4BF	GGACCAGCGTTCCCTGTGC
hATG4BR	CACCAATCATTGAAGTCATC
hGAPDHF	GTCTCCTCTGACTTCAACAGCG
hGAPDHR	ACCACCCTGTTGCTGTAGCCAA
hDNAJC5F	TCCTTGGGTTGGACAAGAAC
hDNAJC5R	TTGAACCCGTCAGTGTGGTA
hITPR3F	GCGGCGCAGATGGAGCAG
hITPR3R	CCGAGGTGGCAGACTGGC
mSORT1F	CAAATGCCAAGG TGGGATGAA
mSORT1R	TTGAATCCAAAGCCTCTACGCC
mATG4BF	GGGCCAACCTTCCCTG
mATG4BR	CACCAGTCGTTAAAGTCTTC
m28SRNAF	GGTTGAGGGCCACCTTATTT
m28SRNAR	GAAGAAAGACCGGGAAGAGAAA

Supplemental Table 1. RT-PCR primers used in this study.

List of primary antibodies used for Western Blotting.

Antibody	Application	Source	Catalogue#	Working dilution
VDAC1	WB	Abcam	ab186321	1:1000
VDAC1	WB	Abcam	ab14734	1:1000
α-Tubulin	WB	Invitrogen	62204	1:5000
GFP	WB	Abcam	ab290	1:1000
GFP	WB	Santa Cruz	sc-9996	1:1000
Flag	WB	Sigma	F3135	1:10000
β-Actin	WB	EMD Millipore	MAB1501	1:10000
MFN2	WB	Abcam	ab56889	1:1000
DRP1	WB	Becton Dickinson Biosciences	611113	1:2000
DRP1 (D6C7)	WB	Cell Signaling Technology	8570	1:1000
MFF	WB	Proteintech	17090-1-AP	1:2000
OXPHOS antibody cocktail-rodent	WB	Abcam	ab110413	1:5000
OXPHOS antibody cocktail-human	WB	Abcam	ab110411	1:5000
CoxIV	WB	Cell Signaling Technology	11967	1:5000
ND3-human	WB	Invitrogen	PA5-103274	1:500
ND6-human	WB	Invitrogen	PA5-109993	1:500
ND3-rodent	WB	Invitrogen	PA5-75180	1:500
ND6-rodent	WB	Invitrogen	PA5-75196	1:500
TDP-43	WB	Proteintech	18280-1-AP	1:1000
TDP-43	WB	Proteintech	10782-2-AP	1:1000
TDP-43	WB	Proteintech	12892-1-AP	1:1000
Human TDP-43	WB	Abnova	H00023435	1:1000
TOM20	WB	Proteintech	11802-1-AP	1:1000
DRP1	WB	Proteintech	26187-1-AP	1:1000
HSP60	WB	Abcam	ab46798	1:2000
GAPDH	WB	Cell Signaling Technology	2118	1:10000

List of primary antibodies used for IHC and IF.

Antibody	Application	Source	Catalogue#	Working dilution
Flag	IF	Sigma	F3135	1:1000
Myc	IF	Cell Signaling Technology	2278	1:100

HB9	IF	Millipore sigma	ABN174	1:50
MAP2	IF	Invitrogen	13-1500	1:300
pTDP409/410	IHC	Proteintech	22309-1-AP	1:50
TDP-43	IHC/IF	Proteintech	10782-2-AP	1:200
TDP-43	IHC/IF	Proteintech	12892-1-AP	1:100
Human TDP-43	IHC/IF	Abnova	H00023435	1:100
NeuN	IHC	Millipore	MAB377	1:1000
GFAP	IHC	Invitrogen	PA5-16291	1:300
Iba1	IHC/IF	Wako	019-19741	1:250
TOM20	IF	Proteintech	11802-1-AP	1:200
SQSTM1	IF	Proteintech	18420-1-AP	1:100
CD68	IF	Abcam	ab125212	1:100
TMEM119	IF	Abcam	ab209064	1:50

List of secondary antibodies used for Western Blotting, IHC, and IF.

Antibody	Application	Source	Catalogue#	Working dilution
Anti-mouse IgG, HRP-linked Antibody	WB	Cell Signaling Technology	7076	1:10000
Anti-rabbit IgG, HRP-linked Antibody	WB	Cell Signaling Technology	7074	1:10000
Goat anti-Rabbit IgG (H+L), Alexa Fluor 488	IF	Invitrogen	A-11008	1:250
Goat anti-Mouse IgG (H+L), Alexa Fluor 488	IF	Invitrogen	A-11001	1:250
Goat anti-Rabbit IgG Alexa Fluor 568	IF	Invitrogen	A-11011	1:250
Goat anti-Mouse IgG Alexa Fluor 568	IF	Invitrogen	A-11004	1:250
Goat anti-Rabbit Alexa Fluor 647	IF	Invitrogen	A-21245	1:250
Goat anti-Mouse Alexa Fluor 647	IF	Invitrogen	A-21236	1:250
SignalStain® Boost IHC Detection Reagent (HRP, Rabbit)	IHC	Cell Signaling Technology	8114	No dilution
SignalStain® Boost IHC Detection Reagent (HRP, Mouse)	IHC	Cell Signaling Technology	8125	No dilution

Supplemental Table 2. Antibodies used in this study. WB: western blot; IF: Immunofluorescence; IHC: Immunohistochemistry.
