# Supplementary material for: Therapeutic targeting of the conserved region within the low-complexity domain of TDP-43 is neuroprotective and extends survival in amyotrophic lateral sclerosis mice
Source: Nat Aging. 2026 Jul 3;6(8):1667–83. doi: 10.1038/s43587-026-01166-3 (PMC13472882; doi:10.1038/s43587-026-01166-3)
Supplement: Supplementary file 3 [file 43587_2026_1166_MOESM2_ESM.pdf]

nature portfolio

Corresponding author(s): Xinglong Wang

Last updated by author(s): Jun 3, 2026

Reporting Summary

Nature Portfolio wishes to improve the reproducibility of the work that we publish. This form provides structure for consistency and transparency in reporting. For further information on Nature Portfolio policies, see our Editorial Policies and the Editorial Policy Checklist.

Statistics

For all statistical analyses, confirm that the following items are present in the figure legend, table legend, main text, or Methods section.

n/a	Confirmed	
☐	☒	The exact sample size (*n*) for each experimental group/condition, given as a discrete number and unit of measurement
☐	☒	A statement on whether measurements were taken from distinct samples or whether the same sample was measured repeatedly
☐	☒	The statistical test(s) used AND whether they are one- or two-sided<br>*Only common tests should be described solely by name; describe more complex techniques in the Methods section.*
☒	☐	A description of all covariates tested
☒	☐	A description of any assumptions or corrections, such as tests of normality and adjustment for multiple comparisons
☐	☒	A full description of the statistical parameters including central tendency (e.g. means) or other basic estimates (e.g. regression coefficient) AND variation (e.g. standard deviation) or associated estimates of uncertainty (e.g. confidence intervals)
☐	☒	For null hypothesis testing, the test statistic (e.g. *F*, *t*, *r*) with confidence intervals, effect sizes, degrees of freedom and *P* value noted<br>*Give P values as exact values whenever suitable.*
☒	☐	For Bayesian analysis, information on the choice of priors and Markov chain Monte Carlo settings
☒	☐	For hierarchical and complex designs, identification of the appropriate level for tests and full reporting of outcomes
☒	☐	Estimates of effect sizes (e.g. Cohen's *d*, Pearson's *r*), indicating how they were calculated

Our web collection on statistics for biologists contains articles on many of the points above.

Software and code

Policy information about availability of computer code

Data collection	Confocal and live-cell imaging data were acquired using a Leica STELLARIS 5 microscope with LAS X v3.7 software (Leica Microsystems). Behavioral data were collected using ANY-maze v7.3 (Stoelting). Mitochondrial respiration data were acquired using DatLab v7 (Oroboros Instruments). Surface plasmon resonance data were collected and processed using BIAevaluation v4.1 (GE Healthcare). RNA sequencing data were generated using the MGI T7 platform and processed using HISAT2 v2.2.1, Bowtie2 v2.3.4.3, and RSEM v1.3.1. LC–MS/MS proteomic data were acquired using a timsTOF Pro2 Q-TOF system and processed using MaxQuant v2.7.3.0. CRISPR sgRNAs were designed using Benchling. Virtual screening and molecular docking were performed using the Schrödinger Maestro suite release 2022-1, including LigPrep, Glide, Epik, QikProp, Protein Preparation Wizard, and Induced Fit Docking workflows.
Data analysis	Image processing and quantitative analyses were performed using Fiji/ImageJ v2.9, Imaris 7 (Oxford Instruments), Image-Pro Plus 6.0 (Media Cybernetics), and LAS X v3.7. Transcriptomic analyses were performed using DESeq2 v1.4.5, DEGseq, and PoissonDis. Proteomic downstream analyses were conducted using Perseus v2.0.10.0. Statistical analyses and graphical visualization were performed using GraphPad Prism 8 (GraphPad Software).

For manuscripts utilizing custom algorithms or software that are central to the research but not yet described in published literature, software must be made available to editors and reviewers. We strongly encourage code deposition in a community repository (e.g. GitHub). See the Nature Portfolio guidelines for submitting code & software for further information.

Data

Policy information about availability of data

All manuscripts must include a data availability statement. This statement should provide the following information, where applicable:

- Accession codes, unique identifiers, or web links for publicly available datasets
- A description of any restrictions on data availability
- For clinical datasets or third party data, please ensure that the statement adheres to our policy

The mass spectrometry proteomics data generated in this study have been deposited in the ProteomeXchange Consortium via the PRIDE partner repository under accession code PXD060626. Bulk RNA-seq data have been deposited in the Sequence Read Archive (SRA) under accession code PRJNA1220042. Source data underlying graphs and charts are provided with this paper. All other data supporting the findings of this study are available within the paper and its Supplementary Information or from the corresponding author upon reasonable request. No custom code was generated in this study.

Research involving human participants, their data, or biological material

Policy information about studies with human participants or human data. See also policy information about sex, gender (identity/presentation), and sexual orientation and race, ethnicity and racism.

Reporting on sex and gender	n/a
Reporting on race, ethnicity, or other socially relevant groupings	n/a
Population characteristics	n/a
Recruitment	n/a
Ethics oversight	n/a

Note that full information on the approval of the study protocol must also be provided in the manuscript.

Field-specific reporting

Please select the one below that is the best fit for your research. If you are not sure, read the appropriate sections before making your selection.

☒ Life sciences ☐ Behavioural & social sciences ☐ Ecological, evolutionary & environmental sciences

For a reference copy of the document with all sections, see nature.com/documents/nr-reporting-summary-flat.pdf

Life sciences study design

All studies must disclose on these points even when the disclosure is negative.

Sample size	Sample sizes were determined based on prior experience with similar assays and power analysis to detect a 20% difference with $\alpha = 0.05$ and power = 0.8. Exact n values are provided in the figure legends.
Data exclusions	No data were excluded from the analyses unless samples failed predefined technical quality-control criteria, such as failed tissue collection, failed transfection/infection, poor staining quality, or imaging artifacts.
Replication	Experiments were independently repeated at least three times with similar results unless otherwise indicated. Technical replicates were not treated as independent biological replicates.
Randomization	No method of randomization was used to assign animals or samples to experimental groups.
Blinding	Behavioral assessments were performed by investigators blinded to treatment allocation and genotype. Other experiments and analyses were not performed under blinded conditions unless otherwise stated.

Reporting for specific materials, systems and methods

We require information from authors about some types of materials, experimental systems and methods used in many studies. Here, indicate whether each material, system or method listed is relevant to your study. If you are not sure if a list item applies to your research, read the appropriate section before selecting a response.

Materials & experimental systems

n/a	Involved in the study
☐	☒ Antibodies
☐	☒ Eukaryotic cell lines
☒	☐ Palaeontology and archaeology
☐	☒ Animals and other organisms
☒	☐ Clinical data
☒	☐ Dual use research of concern
☒	☐ Plants

Methods

n/a	Involved in the study
☒	☐ ChIP-seq
☒	☐ Flow cytometry
☒	☐ MRI-based neuroimaging

Antibodies

Antibodies used

Primary antibodies used for western blotting included VDAC1 (Abcam, ab186321 and ab14734, 1:1,000), α-tubulin (Invitrogen, 62204, 1:5,000), GFP (Abcam, ab290, 1:1,000; Santa Cruz Biotechnology, sc-9996, 1:1,000), Flag (Sigma, F3135, 1:10,000), β-actin (EMD Millipore, MAB1501, 1:10,000), MFN2 (Abcam, ab56889, 1:1,000), DRP1 (BD Biosciences, 611113, 1:2,000; Cell Signaling Technology, 8570, 1:1,000; Proteintech, 26187-1-AP, 1:1,000), MFF (Proteintech, 17090-1-AP, 1:2,000), OXPHOS antibody cocktails (Abcam, ab110413 and ab110411, 1:5,000), CoxIV (Cell Signaling Technology, 11967, 1:5,000), ND3 (Invitrogen, PA5-103274 and PA5-75180, 1:500), ND6 (Invitrogen, PA5-109993 and PA5-75196, 1:500), TDP-43 (Proteintech, 18280-1-AP, 10782-2-AP, and 12892-1-AP, 1:1,000), human TDP-43 (Abnova, H00023435, 1:1,000), TOM20 (Proteintech, 11802-1-AP, 1:1,000), HSP60 (Abcam, ab46798, 1:2,000), and GAPDH (Cell Signaling Technology, 2118, 1:10,000).Primary antibodies used for immunohistochemistry and immunofluorescence included Flag (Sigma, F3135, 1:1,000), Myc (Cell Signaling Technology, 2278, 1:100), HB9 (MilliporeSigma, ABN174, 1:50), MAP2 (Invitrogen, 13-1500, 1:300), pTDP409/410 (Proteintech, 22309-1-AP, 1:50), TDP-43 (Proteintech, 10782-2-AP, 1:200; 12892-1-AP, 1:100), human TDP-43 (Abnova, H00023435, 1:100), NeuN (Millipore, MAB377, 1:1,000), GFAP (Invitrogen, PA5-16291, 1:300), Iba1 (Wako, 019-19741, 1:250), TOM20 (Proteintech, 11802-1-AP, 1:200), SQSTM1 (Proteintech, 18420-1-AP, 1:100), CD68 (Abcam, ab125212, 1:100), and TMEM119 (Abcam, ab209064, 1:50). Secondary antibodies included HRP-linked anti-mouse and anti-rabbit antibodies (Cell Signaling Technology, 7076 and 7074, 1:10,000), Alexa Fluor-conjugated secondary antibodies (Invitrogen, 1:250), and SignalStain Boost IHC Detection Reagents (Cell Signaling Technology, 8114 and 8125). Antibody validation information was based on manufacturer datasheets, previous literature, and/or experimental controls where applicable.

Validation

All primary antibodies used in this study are listed in Supplementary Table 2, including vendor information, catalog numbers, and validation information based on manufacturer datasheets, prior publications, knockout controls, and/or expected molecular weights or staining patterns.

Eukaryotic cell lines

Policy information about cell lines and Sex and Gender in Research

Cell line source(s)

HEK293 cells were obtained from American Type Culture Collection (ATCC).

Authentication

Cell lines were authenticated by the suppliers. HEK293 cells were routinely monitored for morphology and growth characteristics.

Mycoplasma contamination

All cell lines used in this study tested negative for mycoplasma contamination.

Commonly misidentified lines (See ICLAC register)

HEK293 cells used in this study are not listed in the ICLAC database of commonly misidentified cell lines.

Animals and other research organisms

Policy information about studies involving animals; ARRIVE guidelines recommended for reporting animal research, and Sex and Gender in Research

Laboratory animals

Wild-type C57BL/6J mice (strain#000664, Jackson Laboratory) and Prnp-TARDBPA315T transgenic mice (strain#010700, Jackson Laboratory) on a C57BL/6 background were used. Mouse age, sex, and sample size for each experiment are provided in the Methods, figure legends, and reporting summary.

Wild animals

N/A

Reporting on sex

Both genders were included.

Field-collected samples

N/A

Ethics oversight

Mouse surgery and procedures were performed according to NIH guidelines and were approved by the Institutional Animal Care

Note that full information on the approval of the study protocol must also be provided in the manuscript.

Plants

Seed stocks

Report on the source of all seed stocks or other plant material used. If applicable, state the seed stock centre and catalogue number. If plant specimens were collected from the field, describe the collection location, date and sampling procedures.

Novel plant genotypes

Describe the methods by which all novel plant genotypes were produced. This includes those generated by transgenic approaches, gene editing, chemical/radiation-based mutagenesis and hybridization. For transgenic lines, describe the transformation method, the number of independent lines analyzed and the generation upon which experiments were performed. For gene-edited lines, describe the editor used, the endogenous sequence targeted for editing, the targeting guide RNA sequence (if applicable) and how the editor was applied.

Authentication

Describe any authentication procedures for each seed stock used or novel genotype generated. Describe any experiments used to assess the effect of a mutation and, where applicable, how potential secondary effects (e.g. second site T-DNA insertions, mosiacism, off-target gene editing) were examined.
